# Imaging of macrophage accumulation in solid tumors with ultrasound

**DOI:** 10.1038/s41467-025-61624-1

**Published:** 2025-07-09

**Authors:** Ashley Alva, Chulyong Kim, Pranav Premdas, Yann Ferry, Hohyun Lee, Nidhi Lal, Bowen Jing, Edward Botchwey, Brooks D. Lindsey, Costas Arvanitis

**Affiliations:** 1 Electrical and Computer Engineering, Georgia Institute of Technology, Atlanta, GA USA; 2 Woodruff School of Mechanical Engineering, Georgia Institute of Technology, Atlanta, GA USA; 3 Coulter Department of Biomedical Engineering, Georgia Institute of Technology and Emory University, Atlanta, GA USA

**Keywords:** Monocytes and macrophages, Ultrasound, Cancer imaging

## Abstract

Imaging macrophage trafficking in solid tumors has major implications for cancer diagnosis, prognosis, and therapy. Here, we show that macrophage labeling with lipid-shelled microbubbles enables ultrasound imaging at single-cell level. Crucially, microbubble labeling and sonication at low mechanical indexes do not affect macrophage viability, migration, phenotype, and cytokine secretion profile, supporting the notion that ultrasound imaging can be used for nondestructive macrophage imaging. Despite the damping exerted on the microbubble oscillations by the cellular compartments, the microbubbles exhibit highly nonlinear behavior upon sonication, allowing for high specificity nonlinear US imaging under in vitro and in vivo conditions. Subsequently, we demonstrate that nonlinear ultrasound imaging can selectively monitor macrophage accumulation and extravasation in solid tumors in rodents for at least 8 h after intravenous administration. These findings establish ultrasound as a noninvasive platform for immune cell trafficking in solid tumors and highlight its potential to advance cancer diagnosis, monitoring, and therapy.

## Abstract (web summary)

Imaging macrophage trafficking in solid tumors has implications for cancer diagnosis and prognosis. by labeling macrophages with lipid-shelled microbubbles in combination with ultrasound, the authors here achieve nondestructive in vivo intravenously administrated macrophage imaging at single cell level with 100 µm resolution till 8 h in solid tumors in rodents.

## Introduction

Imaging of macrophage trafficking in solid tumors can provide critical information about cancer progression and response to therapy^1,2^. Most notably, high density of M2-like macrophages in the tumor microenvironment (TME) is a robust marker of poor survival in many solid tumors (e.g., breast cancer)^1,2^, as such macrophage (M2-like) accumulation and distribution in tumors is rapidly becoming an important prognostic biomarker^3^. While high M2-like macrophage accumulation reflects a highly dysregulated TME (i.e., pro-tumorigenic inflammation)^4^, M1-like macrophages have been linked to improved overall survival^5,6^. Besides their prognostic value, these observations have also inspired a range of cancer immunotherapy strategies, including chimeric antigen receptor macrophages^7^, that aim to modulate the macrophage state and function to promote antitumor immunity^8,9^. These strategies take advantage of the macrophages ability to (1) penetrate solid tumors and metastasis, even when administered intravenously^7,10^, (2) directly attack cancer cells (e.g., via phagocytosis)^7^, and (3) promote adaptive anti­tumor immune responses (i.e., via antigen presentation)^7^. Moreover, treatments, such as chemotherapy and radiotherapy, can affect macrophage density and influx in solid tumors in different ways^11^. For instance, following radiotherapy there is an increase in relative macrophage density in the tumor that has also been associated with dynamic bursts of chemotherapy extravasation^12^, indicating that even short-term macrophage tracking can lead to the extraction of biomarkers related to improved chemotherapy delivery. Together, these discoveries, apart from demonstrating the diagnostic, prognostic, and therapeutic potential of macrophages, also underscore the importance and potential opportunities created by clinically relevant methods to track and quantify their trafficking patterns (e.g., flux, density).

The recent developments in macrophage tracking methods have been significant and at the same time unsatisfactory^13^. Most notably, optical techniques allow parsing macrophage trafficking at cellular resolution^11,14^, however they do not scale very well, which limits their use to preclinical investigations or superficial organs. MRI provides high resolution imaging along with detailed anatomical information^15^, but faces formidable tradeoffs between specificity (high for ^19^F-labeled cells) and sensitivity (high for SPIO-labeled cells), with the current in vivo detection limit being of the order of 10^4^ cells (or 10^6^ cells/ml)^13,16^. PET can increase the sensitivity limit by more than one order of magnitude^17,18^, however it is hampered by low resolution (spatial and temporal), limited anatomic detail, and dosimetry considerations^19^. Finally, CT offers whole body imaging capability but has low sensitivity and requires high doses of radiopaque agents^13,20^. While the imaging tradeoffs offered by current cell tracking methods could potentially be reconciled by multimodality imaging, such approaches come at increased complexity, cost, and logistical burden^21^. Hence, macrophage tracking methods with more balanced tradeoffs between cell detection specificity, sensitivity, resolution, and penetration depth without adding operational complexity have the potential to both support and accelerate the discovery and translation to the clinic of macrophage-based diagnostic and therapeutic interventions.

Ultrasound (US) can potentially provide a viable solution towards addressing these cell tracking challenges, as US systems are scalable, relatively cheap, portable (e.g., used in an outpatient setting), and routinely used in the clinic when real-time dynamic imaging is needed^22^, which is critical for cell tracking. Yet, there is a paucity of investigations using this imaging modality for macrophage imaging^3^. This is because the inherently low compressibility and density differences of the macrophages with the host cells makes their scattering cross-section comparable (e.g., ${\sigma }_{\mathrm{macrophage}}\approx {\sigma }_{\mathrm{cancer}}$)^22,23^ and as a result they produce poor image contrast. To overcome the underlying limitations of US imaging and enhance its contrast, it has recently been proposed to label the macrophages with superheated nanodroplets and image them by US following US induced nanodroplet vaporization (i.e., phase transition from liquid to gas)^24,25^. While improved image contrast has been attained using this method, nanodroplet imaging is destructive, which limits its ability to perform cell tracking. Labeling the macrophages with microbubble (MB) ultrasound contrast agents, on the other hand, can improve their effective scattering cross-section by several orders of magnitude^22^. This level of improvement may also mitigate the need for destructive imaging (i.e., bursting the MB), which is critical for assessing cell trafficking patterns in vivo. Moreover, due to the inherent nonlinear behavior of MBs (i.e., nonlinear MB oscillation generates harmonics of the imaging frequency), they can potentially be tracked with very high specificity using dedicated US pulse sequencies, allowing them to be discriminated by (filtering out) the primarily linear background^26^.

**Fig. 1:**
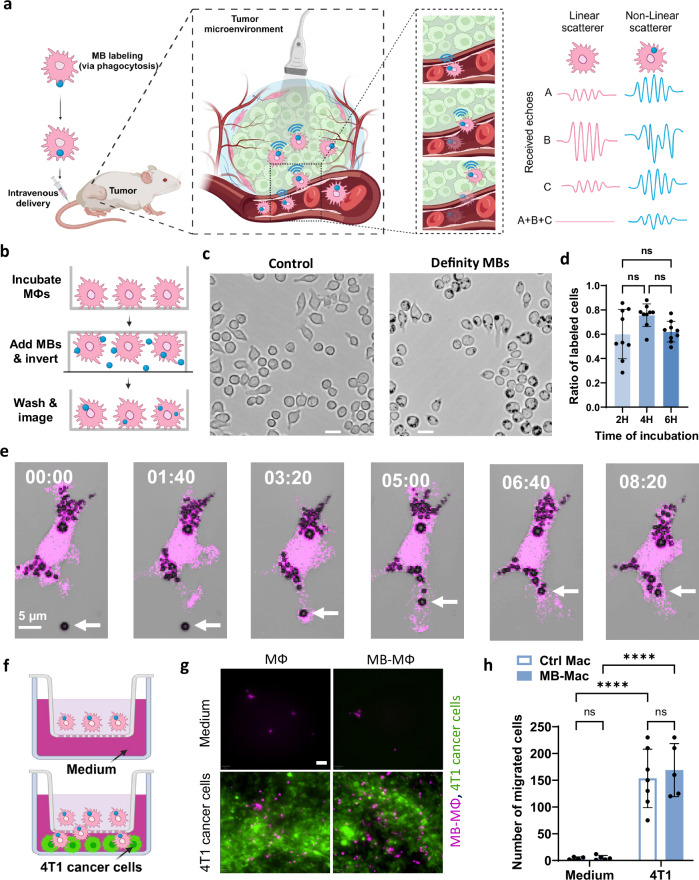
Macrophages can be labeled with microbubbles with high efficiency and without affecting their migration capability. **a** Conceptual depiction of the proposed approach to track macrophage trafficking with ultrasound imaging. “Created in BioRender. Kim (2025) https://BioRender.com/l88d5ia, https://BioRender.com/s2lkzzu”. **b** Schematic showing the setup and protocol for labeling RAW264.7 macrophages (MΦ) with microbubbles (MB). “Created in BioRender. Kim (2025) https://BioRender.com/bcw4jei”. **c** Representative microscopy images of MΦ following 4 h of incubation with and without MBs (black dots). Scale bar 20 µm. See Supplementary Movie 1. **d** Quantification of MΦ-labeling with MBs at different incubation (*n* = 9 wells). Data are presented as mean values ± SD. *p* values were determined by one-way ANOVA, n.s. not significant. **e** Panel of live confocal microscopy images illustrating MBs-uptake by macrophages (magenta: mCherry). **f** Schematic of the Boyden chamber with and without cancer cells (created with BioRender.com. “Created in BioRender. Kim (2025) https://BioRender.com/f4irfqi”. **g** Representative fluorescent microscopy images from the Boyden chamber of the 4T1 cancer cells (green) and MB-MФ and MФ (pink). Scale bar 50 μm. **h** Quantitative analysis of the number of migrated MΦ and MB-MΦ in presence and absence of 4T1 cells (*n* = 4 wells). Data are presented as mean values ± SD. *p* values were determined by two-way ANOVA followed by Tukey’s multiple comparisons.****p < 0.0001; n.s. not significant. (*p* value for Ctrl Mac and MB-Mac with 4T1 is *p* = 0.9137 and Ctrl Mac and MB-Mac in Medium is *p* > 0.9999). Source data are provided as a Source Data file.

Hence, we hypothesize that labeling macrophages with MB US contrast agents can overcome the inherent limitations of US and enable imaging of macrophage trafficking with high sensitivity and specificity while retaining high resolution (Fig. 1a). To test this hypothesis, we first develop methods to effectively label macrophages using MBs by taking advantage of the inherent ability of macrophages to phagocytose micron scale particles. We then assess the impact of phagocytosis on MB oscillation amplitude along with the impact of MB labeling and US exposure on macrophage migration capabilities, phenotype and cytokine secretion profile, and cell viability, respectively. Next, we determine the detection limit of MB-labeled macrophages using both linear and nonlinear ultrasound imaging methods under in vitro and in vivo conditions and assess their capabilities for image-guided cell infusion procedures using real-time dynamic US imaging. Finally, we assess the accumulation of intravenously administered MB-labeled macrophages in breast tumor-bearing mice using linear and nonlinear US imaging as well as flow cytometry and fluorescence microscopy. Together our findings demonstrate the potential of MB-labeled macrophages in combination with ultrasound imaging to provide a noninvasive, portable, and scalable platform to study the complex problem of immune cell trafficking in solid tumors.

## Results

### Macrophages can be robustly labeled with microbubbles without losing their migration capabilities or altering their phenotype

First, we developed experimental methods and protocols to promote and study MB uptake by macrophages (from now on MФ). Briefly, after culturing MФs on a petri dish, we introduced MBs (${10}^{10}$ bubbles/ml) into the medium and inverted the petri dish to increase their contact with the MФs, by taking advantage of MB buoyancy forces, and facilitate labeling via phagocytosis (Fig. 1b). In this proof of concept investigation, we employed MФ cell line RAW264.7, which is a frequently used cell line for testing new MФ imaging methods^14^, as well as primary bone marrow derived macrophages from mice, primary human macrophages, and human dendritic cells. For the MBs, we employed the U.S. Food and Drug Administration-approved formulation, Definity (Lantheus Medical Imaging)^27^. After washing out excess MBs, we observed that the RAW264.7 MФs have been labeled successfully following 2 (60.0% ± 20.3%), 4 (75.7% ± 9.3%) or 6 (62.1% ± 8.5%) hours of co-incubation with Definity MBs (Fig. 1c, d). Live cell microscopy allowed us to record the engulfment of a microbubble by a macrophage and estimate the time necessary for the phagocytosis of a single microbubble, which can be as low as 10 min (Fig. 1e and Supplementary Movie 1). Having established a protocol for robust labeling, we assessed if the MB-labeling impacted the migration capability of the MФs, using the transwell migration assay (Fig. 1f). To facilitate migration, we cultured 4T1 cancer cell lines in the layer below the cell permeable layer (i.e., MФs were placed on top of this layer), as they can be very potent in attracting the RAW264.7 MФs^24^. Indeed, in the absence of 4T1 breast cancer cells, we observed marginal migration of MФs or MB-MФs across the membrane, however, in the presence of 4T1 cancer cells both MФs or MB-MФs migrated at the lower level with similar efficiency, suggesting that MB labeling does not affect this key function of the MФs (Fig. 1g, h).

**Fig. 2:**
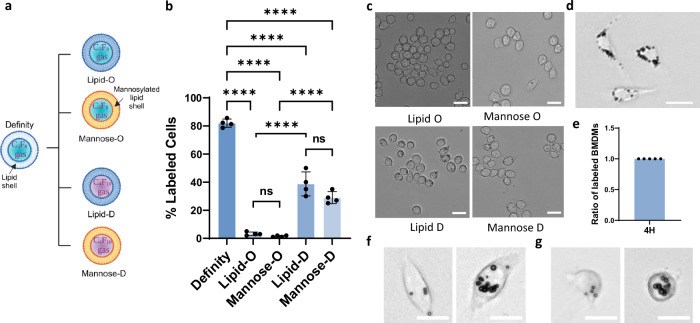
Microbubbles compositions and cell line influence microbubble uptake. **a** Schematic representation of the different MB composition that we incubated with MΦs. “Created in BioRender. Kim (2025) https://BioRender.com/kqv3lyu”. **b** Quantification and **c** representative microscopy images of MΦ-labeling with Lipid MBs with different shell composition (mannose shell vs. lipid shell) and gas core (Octafluoropropane—C3F8—shown with “O” vs. Decafluorobutane—C4F10—shown with “D”) developed in house following 4 h of incubation. Scale bar 20 μm. (*n* = 4 petri dishes per group). Data are presented as mean values ± SD. *p* values were determined by one-way ANOVA followed by Tukey’s multiple comparisons. ****p < 0.0001; n.s. not significant. (*p* value for Lipid-O and Mannose-O is *p* = 0.9805 and Lipid-D and Mannose-D is *p* = 0.0533). **d** Representative microscopy images of MB-BMDMs. Scale bar 20 μm. **e** Quantification of MB uptake by BMDMs (*n* = 5 animals). Representative microscopy images of primary human macrophages (**f**) and dendritic cells (**g**) labeled with microbubbles. Scale bar 20 μm. Similar results were observed over three different blood samples for human macrophages and two different blood samples for dendritic cells. Source data are provided as a Source Data file.

Then, we examined how MB properties influence their uptake by MФs. We fabricated lipid MBs with different gas and shell properties (Fig. 2a) and found that lipid-MBs with a lower diffusivity gas (C4F10), which leads to higher MB stability, were taken up more avidly than those with higher diffusivity gas (C3F8). While this might seem to contradict our findings with the Definity MBs, which are made of the lower diffusivity gas (C3F8)^28^ (Fig. 2b, c), Definity MBs had much higher stability on the petri dish as compared to the MBs we fabricated. This suggests that MB stability, rather than gas content, is critical for promoting MB uptake by MФs. We also tested the impact of MB shell on MФ labeling by employing a mannosylated lipid shell, but we found similar trends, indicating that RAW264.7 MФs do not preferentially phagocytose mannose microparticles.

To corroborate the translational potential of our method, we assessed our labeling protocol with primary bone-marrow derived macrophages (BMDMs), as well as primary human macrophages and dendritic cells (see “Methods”). We confirmed the appropriate differentiation of these cells based on surface marker expression (CD11b and F4/80 for BMDMs and CD14, CD11b, CD68, and CD1a for human macrophages and dendritic cells; see Supplementary Figs. 1 and 2). Using the same labeling protocol applied to RAW264.7 MФs, we observed strong MB uptake across all types, with 100% uptake observed in BMDMs after 4 h of coincubation with Definity MBs (Fig. 2d, e), and similarly high uptake in both human macrophages (Fig. 2f) and dendritic cells (Fig. 2g), supporting the robustness and clinical relevance of our methods.

**Fig. 3:**
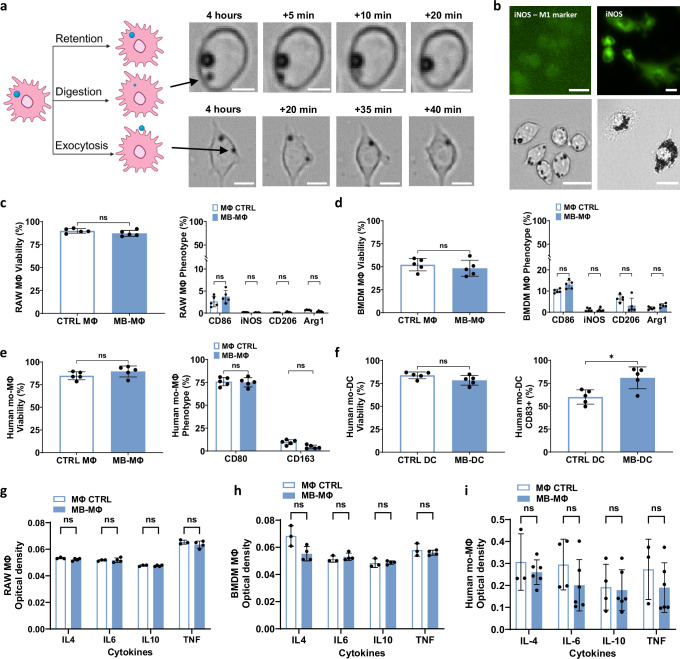
Microbubble labeling is impacted by macrophage activity and polarization but does not impact macrophage polarization. **a** Microscopy images showing different fates of MBs once phagocytosed—Retention, Digestion, Exocytosis. Scale bar 10 µm. “Created in BioRender. Kim (2025) https://BioRender.com/shd7dvr”. **b** Top left: iNOS stained untreated MΦs, bottom left: representative image of microbubbles uptake by untreated MΦs, top right: iNOS stained MΦs polarized to M1, bottom right: representative image of microbubbles uptake by M1 MΦs. The MФs were polarized with LPS (100 ng/ml) and IFN-γ (20 ng/ml). Scale bar 20 µm. Flow cytometry of cell viability (left) and expression of markers reflecting phenotype changes (right) following MB labeling in **c** RAW264.7 cell lines, **d** BMDM mice cells, **e** primary human macrophages, and **f** primary human DCs. (*n* = 5 wells for RAW264.7, *n* = 5 animals for BMDMs, *n* = 5 wells for human macrophages and DC). Data are presented as mean values ± SD. *p* values were determined by using two tailed unpaired *t*-test **c** left, **d** left, **e** left and **f** or a two-way ANOVA followed by Tukey’s multiple comparisons **c** right, **d** right, **e** right. n.s. not significant. **g**–**i** Optical density read by ELISA assay of IL4, IL6, IL 10, and TNF released in the 24 h following RAW264.7 macrophages, BMDMs and human macrophages labeling compared to non-labeled. (*n* = 3 wells for RAW264.7, *n* = 3 mice for BMDMs, *n* = 4 wells for human macrophages). Data are presented as mean values ± SD. *p* values were determined by using a two-tailed unpaired *t*-test with Holm-Šídák correction; n.s. not significant. Source data are provided as a Source Data file.

Next, using live cell microscopy, we assessed the MB fate inside MФs (RAW264.7) following 4 h of co-incubation. We observed that, in addition to being retained within the MФ, MBs can also be digested or undergo exocytosis (Fig. 3a). We further assessed the impact of macrophage phenotype on MB uptake by polarizing the MФs towards M1 phenotype using lipopolysaccharide (LPS) and IFN-γ^29^, and monitored the MB uptake after 4 h of co-incubation (Fig. 3b). Interestingly, M1-like MФs demonstrated a dramatic increase in bubble uptake as compared to unpolarized MФs, suggesting that MФ-phenotype can be tuned to modulate the MB labeling.

We also investigated whether MB uptake by different cell types including RAW264.7 cells, BMDMs, and primary human macrophages and dendritic cells could lead to phenotype changes (e.g., M1/M2-like states, or activation/maturation) in vitro. Flow cytometry analysis (Fig. 3c–f) revealed no significant changes in the expression of CD86, iNOS, CD206, or Arg1 in RAW264.7 and BMDMs, nor in CD80 and CD163 for human macrophages, indicating that MB labeling does not alter the phenotype of these cell types. However, in primary human dendritic cells, we observed a significant increase in CD83 expression (*p* < 0.05) in the MB-labeled group, suggesting that phagocytosis of MBs may promote mild maturation or activation in DCs. To assess labeling-related toxicity, we also measured cell viability across all cell types. We observed no significant reduction in viability between MB-labeled and non-labeled groups (Fig. 3c–f), confirming the biocompatibility of the labeling method and the lack of MB-related toxicity across all tested cells. To further investigate the effect of MB-uptake on MΦs, we measured the cytokines secreted by RAW264.7 cells, BMDMs and human macrophages 24 h following MB labeling. We observed no significant difference (Fig. 3g–i) between labeled and non-labeled groups, indicating that MB uptake does not trigger an inflammatory response. Note that our positive controls, consisting of M1- and M2-polarized macrophages, showed marked increases in cytokine secretion (see Supplementary Figs. 3 and 4), validating assay’s performance. We also confirmed the ability of MB-BMDMs to migrate in the presence of a chemoattractant (see Supplementary Fig. 5). Despite the critical role of polarization and the dynamic process that characterizes MB phagocytosis, phagocytosed MBs remain within the macrophages (M0) for several hours (Fig. 1d), providing the wide time window that is needed for tracking MФ trafficking. In aggregate, these findings demonstrate that it is possible to label MФ with the FDA-approved MB formulation, Definity, via phagocytosis for several hours without affecting their migration capabilities or altering their phenotype.

### Phagocytosed microbubbles remain acoustically active without affecting macrophage viability

**Fig. 4:**
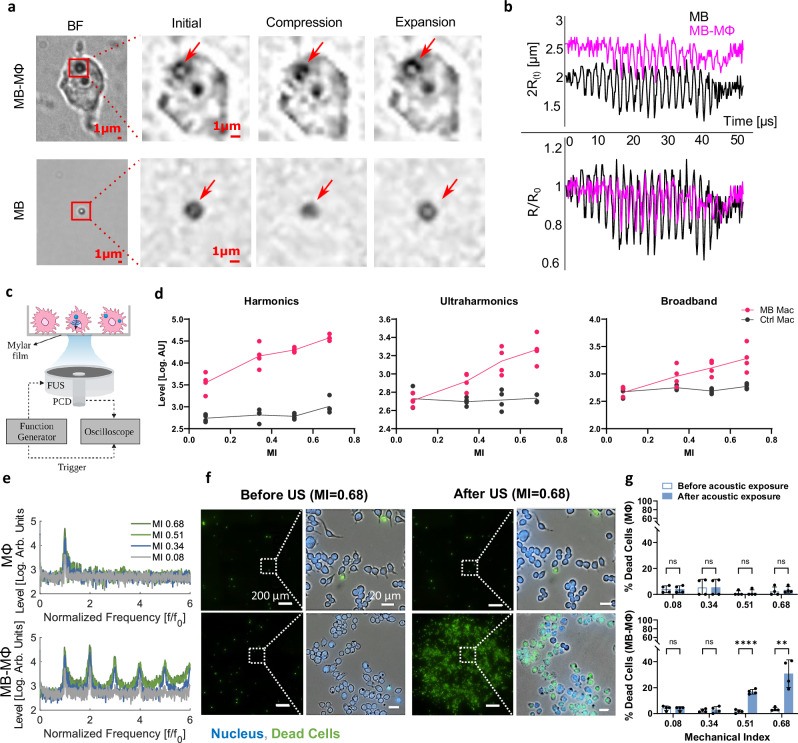
Optical and acoustical characterization of MB-MΦ oscillation reveals damped, compression only, nonlinear MB oscillations for low mechanical indexes without affecting MΦ (RAW264.7) viability. **a** Snapshots from high frame rate microscopy (5 million frames/s) of phagocytosed MBs and unlabeled MBs sonicated at MI = 0.21 (arrows indicate microbubbles). To be able to capture the MB dynamics we excited them at 0.5 MHz. **b** Quantification of high frame rate microscopy data (**a**) revealing phagocytosed MBs display damped, compression-only oscillations. **c** Setup to test echogenicity and viability of MB-MΦ and MΦ. “Created in BioRender. Kim (2025) https://BioRender.com/3ccb31q”. **d** Quantification of the MB-MΦ and MΦ acoustic emissions spectrum for different MIs (*n* = 4 petri dish). The connecting line intersects at the mean. **e** MB acoustic emissions spectrum for MB-labeled and unlabeled MΦ at different MIs. The US pulse duration in these experiments was 20 cycles. **f** Microscopy imaging and **g** quantification of MΦ viability staining before and after sonication at different pressures. Green represents all the non-viable cells and blue represents all cells (viable and non-viable). Top panel: when only unlabeled MΦs are present, showing that even at the highest MIs there is no change in viability. At these conditions only the fundamental frequency is observed at the spectrum. Bottom panel: when the MB-MΦs are present. At MIs above 0.34 the sharp rise in cell death coincides with the onset of broadband emissions in the acoustic emissions. At MIs below 0.34 and as low as 0.08 strong harmonics are present in the acoustic emissions spectrum. (*n* = 4 ROIs). Data are presented as mean values ± SD. *p* values were determined by using a two-tailed unpaired *t*-test with Holm-Šídák correction; **p < 0.01, ****p < 0.0001, n.s. not significant. (MΦ: *p* value for all MI > 0.99 except MI = 0.68 where *p* value = 0.97; MB-MΦ: MI = 0.08, *p* value = 0.89; MI = 0.34, *p* value = 0.89; MI = 0.51, *p* value = 0.00004; MI = 0.68, *p* value = 0.006). Source data are provided as a Source Data file.

Subsequently, we assessed the impact of phagocytosis on MB oscillation as well as the impact of MB oscillation on cell viability. For the former, we employed high frame rate optical microscopy that allows us to directly assess the MB dynamics. We observed that the oscillation of the phagocytosed MB was smaller, as compared to non-phagocytosed bubbles (Fig. 4a) and characterized by compression only behavior (Expansion = 1.6% ± 3.6% vs. 8.2% ± 2.9% and Compression = −19% ± 3.5% vs. −30% ± 5.2%; Fig. 4b, see also Supplementary Movie 2), suggesting that the viscoelastic properties of the surrounding cellular compartment exert significant damping on the phagocytosed MB. Subsequently, using acoustic methods (Fig. 4c), we confirmed that the phagocytosed MBs demonstrated strongly nonlinear behavior (i.e., strong harmonic emissions) when exposed to ultrasound (i.e., sonicated) at Mechanical Indexes (MIs) as low as 0.08 (Fig. 4d, e). Importantly, at exposures where no broadband emissions were observed (MI < 0.34), which indicate stable MB oscillation, the percentage of dead MB-MФs and MФs was comparable (3.3% ± 2.5%). However, in the presence of broadband emissions, the percentage of dead MB-MФs was significantly higher as compared to unlabeled MФs (23.8% ± 6.2% vs. 3.3% ± 2.5%; *p* < 0.0001, Fig. 4f, g). Together, these findings demonstrate that despite the damping exerted to the MB from the surrounding cellular compartments, there is a pressure window that allows to retain and maintain viable (i.e., safe: MI < 0.3) and acoustically active (i.e., generating strongly nonlinear oscillations) MB-MФ.

### Low mechanical index ultrasound imaging can detect as low as one microbubble-labeled macrophage in vitro

**Fig. 5:**
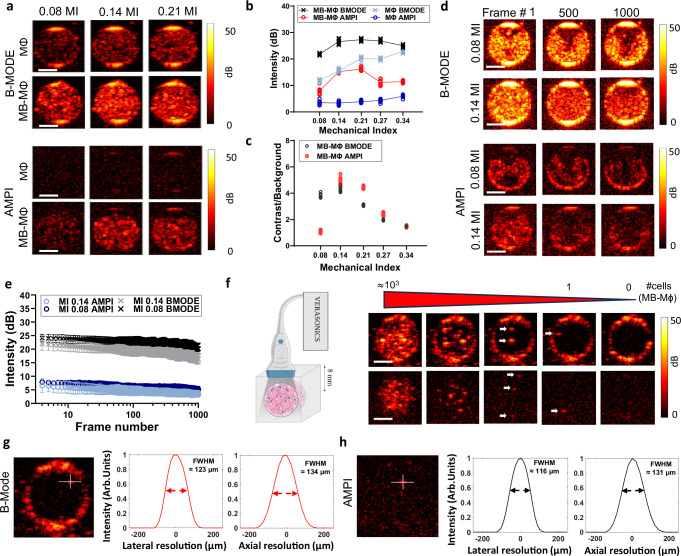
In vitro characterization of the US imaging performance of microbubble-labeled macrophages using linear and nonlinear imaging*.* **a** The panels show a cross-sectional US image of the vessel phantom with the US array arranged perpendicular to the flow (see also **f**). Representative ultrasound images of MB-MΦs and MΦs under linear B-mode imaging and amplitude modulated pulse inversion non-linear imaging at different MIs. Scale bar 1 mm. **b** Quantification of the MB-MΦ and MΦ image intensity using the two methods at varying MIs (*n* = 10 frames). The connecting line is intersecting at the mean. **c** Quantification of the contrast to background of the MB-MΦs and MΦs using the two methods at varying MIs (*n* = 10 frames). **d** Assessment of MB-MΦ signal persistence at different MIs at no flow conditions. Top: Representative B-Mode images at frame 1, frame 500 and frame 1000 at MIs 0.08 and 0.14; bottom: Representative AMPI images at same frame numbers and MIs as B-Mode. Scale bar 1 mm. **e** Quantification of the image intensity as a function of frame number for the two imaging methods at the two MIs (*n* = 3 petri dishes). Data are presented as mean values ± SD. **f** Assessment of the detection limit of the MB-MΦ for B-Mode and AMPI for different number of cells in the imaging volume (MI = 0.14). In the right most images there is only media flowing through the channels. At the next image (second from last) there is one MB-MΦ within the imaging volume ( ≈ 3.2 µl). “Created in BioRender. Kim (2025) https://BioRender.com/r2f29fq”. **g** Experimental determination of the imaging resolution: FWHM: ~123 µm laterally (diffraction limit: 40.5 µm), 134 µm axially (diffraction limit: 98.5 µm) for B-Mode and **h** ~116 µm laterally (diffraction limit: 40.5 µm), 131 µm axially (diffraction limit: 98.5 µm) for AMPI. US imaging (B-Mode and AMPI) was performed with 15.625 MHz linear array transducer (L22-14v, Vermon) that was controlled by a research US platform (Vantage 256, Verasonics). The imaging depth was 8 mm in all data shown. The array characterization is shown in Supplementary Fig. 8. Scale bar 1 mm. Source data are provided as a Source Data file.

After we established a protocol for labeling MФ with the FDA-approved MB formulation, Definity, and identified the US exposure settings such that the encapsulated MB display strong (nonlinear) oscillations without affecting the MB-MФ viability, we turned our attention to their imaging performance. Here, the RAW264.7 MФs or MB-MФs were made to flow through a wall-less tissue mimicking gelatin phantom at a concentration of ${10}^{6}\,$MФ/ml (Fig. 5). To image the cells, we employed an US imaging array with a central frequency of 15.625 MHz, using B-Mode, a linear US imaging scheme, and a combined amplitude modulation and pulse inversion (AMPI; using even-odd technique shown in Supplementary Fig. 6), which is a dedicated pulse sequence for nonlinear MB imaging^30^. First, we confirmed that MB-MФs can be clearly resolved using both pulse sequences (Fig. 5a–c) at MIs that led to negligible cell death in our previous investigations (MI < 0.34, Fig. 4). Both imaging methods had the best imaging performance at MI = 0.14 with AMPI producing slightly higher image contrast due to its effectiveness in suppressing background (linear) signal (Fig. 5c). At much higher MIs the image performance of either method did not improve any further due to combined effects of signal saturation and increasing background signal. To confirm that we could also image labeled BMDMs we imaged the cells using B-Mode and AMPI with the same imaging probe. While our investigations were not as extensive as with the RAW264.7 macrophages, both imaging methods demonstrated a significant increase in SNR (*n* = 3, *p* value = 0.003 for AMPI and *p* value = 0.0134 for B-Mode) when comparing MB-labeled BMDMs to non-labeled cells (see Supplementary Fig. 7).

Next, we assessed the signal persistence in the two imaging methods, which is critical for tracking MФs trafficking, by determining the number of frames that the MB-MФ signal remained constant under no flow conditions at two different MIs (0.08 and 0.14). Both imaging methods had comparable performance, and the signal declined only slightly for 1000 frames (Fig. 5d, e), demonstrating that at these MIs either imaging method can potentially be used to track macrophages. Finally, under in vitro conditions, we determined the US imaging detection limit using these two pulse sequences (Fig. 5f). We found that both methods can detect as few as one cell. While in B-mode imaging this limit might include false positives (i.e., unlabeled macrophages may also contribute to this detection limit), AMPI, which offers very high specificity (i.e., less false positives—see also Fig. 5a), clearly demonstrates that US imaging can reach the theoretical detection limit of one MB-MФ. Moreover, as evidenced by the images that were collected at lower MB-MФs concentrations, the imaging resolution was close to the diffraction limit for both imaging schemes (Fig. 5g, h). In aggregate, these in vitro findings demonstrate the high detection capabilities of US imaging for detecting and tracking MB-MФs and reveal that US can offer improved tradeoffs between cell detection specificity, sensitivity, and resolution at 8 mm penetration depth.

### Low mechanical index ultrasound imaging can detect ten microbubble-labeled macrophages in vivo

**Fig. 6:**
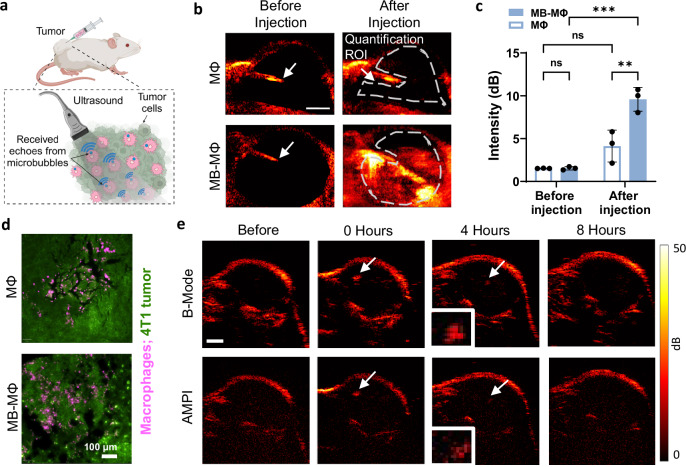
Assessment of signal persistence during US imaging of MB-labeled macrophages in vivo. **a** Schematic of the experimental protocol employed to assess the US imaging performance and detection limit of the MB-MΦ following intratumoral delivery in 4T1 murine breast cancer grown in the flank. “Created in BioRender. Kim (2025) https://BioRender.com/ricv146”. **b** B-mode imaging of intratumoral infusion of MΦs (top) and MB-MΦs (bottom), before injection (left) and after injection using maximum intensity projection (right). White arrow indicates the tip of the syringe used to infuse the MΦs. **c** Image contrast to background ratio before and after the infusion of MΦs (control) and MB-MΦs. (*n* = 3 mice). Data are presented as mean values ± SD. *p* values were determined by two-way ANOVA followed by Tukey’s multiple comparisons. **p < 0.01, ***p < 0.001; n.s. not significant. (MB-MΦ vs. MΦ Before injection, *p* value > 0.9999; MB-MΦ vs. MΦ After injection, *p* value = 0.0019; MΦ Before injection vs. After injection, *p* value = 0.0951; MB-MΦ Before injection vs. After injection, *p* value = 0.0001). **d** Fluorescence microscopy images from tumor sections confirming the delivery of MΦs and MB-MΦs. RAW264.7 MΦs expressing mCherry protein are shown in magenta and 4T1 cancer cells expressing green fluorescent protein are shown in green. **e** Representative B-Mode and AMPI images of 4T1 murine breast cancer before intratumoral MB-MΦ injection, right after injection, 4 h and 8 h later. US imaging (B-Mode and AMPI) was performed with a 15.625 MHz linear array transducer that was controlled by a research US platform (Vantage 256, Verasonics). Source data are provided as a Source Data file.

After identifying the US exposure conditions (MI = 0.14), pulse sequences (B-mode and AMPI) capable of imaging MB-MΦs, and in vitro detection limit (one cell), we sought to test the imaging performance of the proposed macrophage tracking method in vivo. To be able to relate the US image contrast with the number of cells present in the field of view, we directly infused MΦs and MB-MΦs in 4T1 murine breast cancer grown in the flank. The infusion of MB-MΦs was performed under US guidance (i.e. real-time US imaging) (Fig. 6a, Supplementary Fig. 9 and Supplementary Movie 3). First, using a relatively high concentration (10^7^ cells/ml; infused volume: 200 µl), as expected we observed that MB-MΦs led to a significantly higher image contrast (9.9 ± 1.2 dB) as compared to both background (i.e., before MB-MΦ infusion, 2.06 ± 2.00 dB) and non-labeled MΦs (Fig. 6b, c; 1.19 ± 1.28 dB, *p* < 0.001). For the latter, we did not observe a significant change in image contrast (or signal intensity) between pre- and post-infusion imaging, confirming their comparable scattering cross section with the host cells (i.e., ${\sigma }_{\mathrm{macrophage}}\approx {\sigma }_{\mathrm{cancer}}$) and underscoring the need for labeling MΦs to become visible to US in vivo. Interestingly, in a couple of mice (not used in our quantitative analysis), we were also able to track MB-MФs entering the tumor and subsequently exiting the injection site post needle removal (Supplementary Fig. 9 and Supplementary Movie 3), demonstrating the potential of the proposed methods to both track the delivery of macrophages and identify faulty injections. Following US imaging, we harvested the tumors (expressing green fluorescent protein) and confirmed successful MΦ (expressing mCherry protein) delivery using fluorescence microscopy (Fig. 6d). These findings support our hypothesis that labeling macrophages with MBs can enable macrophage imaging with high image contrast in vivo and in solid tumors. To avoid having excessive number of cells in the field of view in subsequent experiments, we reduced the number of infused MB-MΦs by one order of magnitude (10^6^ cells/ml; infused volume: 200 µl). Under these conditions, we observed that the intratumorally infused MB-MФs produced US signal that persisted for at least 4 h post-infusion (Fig. 6e). However, at 8 h post-infusion the MB-MФ population was reduced significantly, presumably due to MB-MФ migration.

**Fig. 7:**
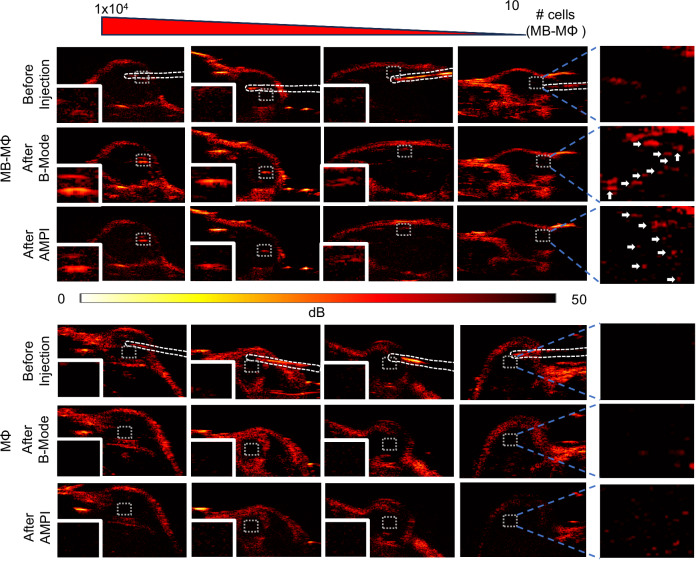
Assessment of the detection limit of MB-labeled macrophages with US imaging under in vivo conditions. Top: Representative B-Mode and AMPI images of different MB-MΦ concentrations ranging from $1\times {10}^{4}$ to 10 with 10-fold reduction in the number of cells. Reference images taken before intratumoral injection of the MB-MΦs are shown on the top panel. Bottom: same image with unlabeled MΦs. The last panel to the right shows the magnified and image enhanced region where the 10 cells were infused. Based on these images the detection limit was determined to be less than 10 MB-MΦs. Scale bar 2 mm. The macrophage infusion was performed using the methods and procedure shown in Fig. 5, Supplementary Fig. 9 and Supplementary Movie 3. US imaging (B-Mode and AMPI) was performed with a 15.625 MHz linear array transducer that was controlled by a research US platform (Vantage 256, Verasonics).

Finally, we sought to determine the detection limit of the MB-MФs. To be able to control the number of administered cells we also directly infused the MB-MФs in the tumors, as we did before (Fig. 6), at different concentrations. Importantly, our analysis indicates that we can detect less than 10 MB-MФs using both imaging methods (Fig. 7). Taken together, our investigations that employed clinically relevant microbubbles (i.e., FDA approved MB ultrasound contrast agent) and imaging methods (B-Mode and AMPI) demonstrated the effectiveness of US for image-guided cell injection procedures and revealed that the in vivo detection limit of MB-MΦ using US is approaching the theoretical limit of one cell.

### Ultrasound imaging can monitor the trafficking of microbubble-labeled macrophages in solid tumors

**Fig. 8:**
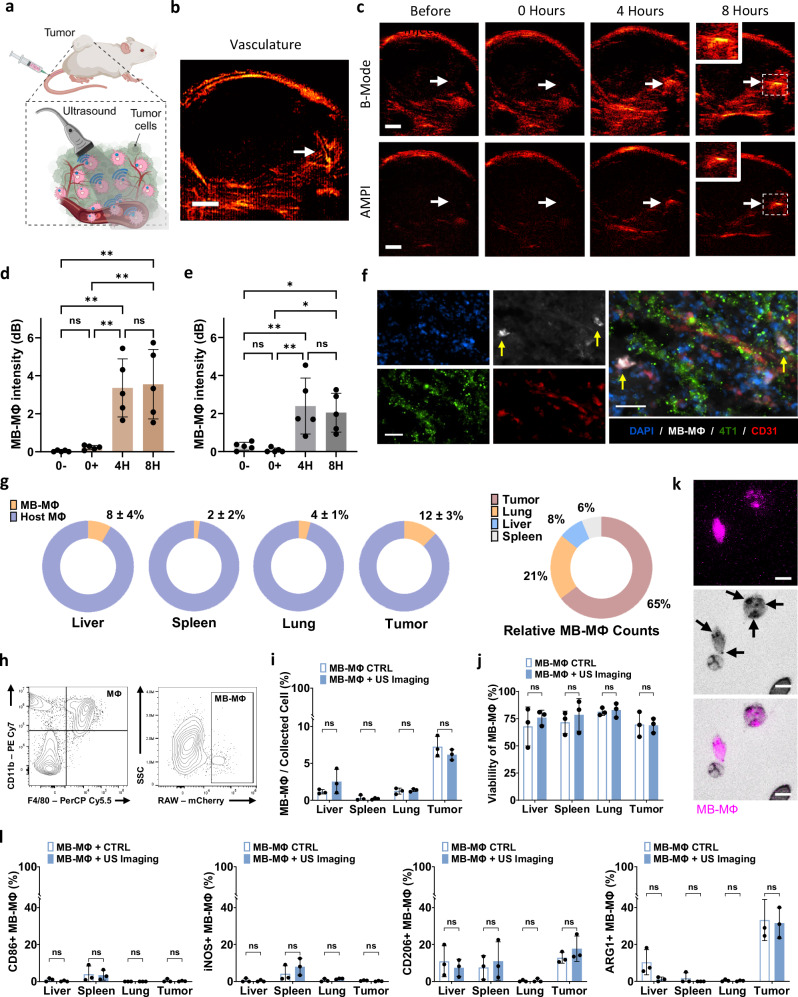
Assessment of the trafficking patterns of intravenously infused MB-labeled macrophages in solid tumors with US. **a** Schematic of the experimental protocol employed to assess MB-MΦ accumulation in 4T1 murine breast cancer with US imaging following intravenous MB-MΦ infusion. “Created in BioRender. Kim (2025) https://BioRender.com/1jad7zr”. **b** Representative image of the tumor vasculature using US imaging following IV MB administration ($5.5\times {10}^{7}$ MBs). In this tumor model the vessels tended to concentrate at the tumor periphery. **c** Representative B-mode and AMPI US images of the tumor before the MB-MФ administration, right after and 4- and 8-h post-administration. Scale bar 2 mm. All the images collected across the tumor are shown in Supplementary Fig. 11. **d**, **e** Quantitative analysis of the B-mode and AMPI image intensity at the 4 different time points (*n* = 5 animals). Data are presented as mean values ± SD. *p* values were determined by a one-way ANOVA followed by Tukey’s multiple comparisons. *p < 0.05, **p < 0.01; n.s. not significant. **f** Fluorescent images showing MB-MФ (magenta) accumulation in the TME (green—cancer cells) 8 h post intravenous administration. Yellow arrow indicates extravasated MФ. Note that there is a small crosstalk between the mcherry (MB-MΦ) and CD31 staining. Scale bar 100 μm. See also Supplementary Fig. 12. **g**–**l** Data from flow cytometry across different organs (liver, spleen, lungs, and tumor) and groups (with and without US imaging). **g** Relative MB-MФ counts across different organs, indicating that about 12% of viable MB-MΦs (compared to the host MΦs) accumulated in the tumor 8 h post intravenous administration. (*n* = 3 animals). **h** Representative gating plots (additional data are shown in Supplementary Fig. 13). **i** Percentage of MB-MФs in the total live cell population collected from each organ. (*n* = 3 animals). Data are presented as mean values ± SD. *p* values were determined by a one-way ANOVA followed by Tukey’s multiple comparisons. n.s. not significant. **j** The viability of the MB-MФs was uniform and above 65% across all organs and groups (i.e., with and without US imaging). (*n* = 3 animals). Data are presented as mean values ± SD. *p* values were determined by a one-way ANOVA followed by Tukey’s multiple comparisons. n.s. not significant. **k** Microscopy images of MB-MФs collected from the disassociated tumor showing that MΦs (pink) retain the MBs (indicated by black arrows in brightfield) for at least up to 8 h. **l** Phenotypic analysis of MB-MΦs from different organs. Data are presented as mean values ± SD. *p* values were determined by a one-way ANOVA followed by Tukey’s multiple comparisons. n.s. not significant. Source data are provided as a Source Data file.

While the data presented so far are critical for assessing the image quality and determining the in vitro/in vivo detection limit of the proposed approach, it is equally important to assess its ability to track macrophage trafficking patterns in tumors following intravenous administration. Therefore, we intravenously infused MB-MФs at a concentration of $2\times {10}^{7}$ cells per ml ($3\times {10}^{6}$ cells in total) and assessed their trafficking in the 4T1 breast tumors using US imaging (Fig. 8a). As before, we employed MI of 0.14 and imaged the tumor using B-Mode and AMPI before the MB-MФ administration, immediately afterwards, as well as 4- and 8-h post-administration. Compared to the experiments with the intratumoral MB-MФ administration, there were two key differences in these experiments. First, to be able to compare the signal at different points, we collected images across the entire tumor and registered the images from the different time points (see Methods Section and Supplementary Figs. 10 and 11). Second, one day before the MB-MФ administration we administered intravenously MBs, which are established vascular contrast agents^22^, and characterized the tumor vasculature using ultrasound localization microscopy methods (see “Methods” section and Supplementary Fig. 10). This allowed us to focus our analysis on MB-MФ trafficking only at these regions where the intravenously administered MB-MФs were expected to infiltrate the tumor. At these regions, which tended to concentrate at the tumor periphery (Fig. 8b), we observed a sharp increase in signal intensity at the 4-h time point that plateaued thereafter for both AMPI and B-Mode (Fig. 8c–e), demonstrating the ability of both linear and nonlinear US imaging to monitor macrophage flux and accumulation in solid tumors. Importantly, fluorescent microscopy of harvested tumors not only confirmed that MB-MФs accumulated in the TME, as indicated by US imaging, but also provided evidence that the MB-MФs were able to extravasate and penetrate the TME (Fig. 8f; see also Supplementary Fig. 12).

To provide a quantitative assessment of the MB-MФs in the TME, we harvested tumors, as well as lungs, spleen, and liver, at 8 h after intravenous infusion and analyzed the MB-MФ counts and viability using flow cytometry. As a preliminary confirmation, microscopy of the single cell suspension from disassociated tumors, prior to cytometry analysis, verified that MΦs retained the MBs for at least up to 8 h (Fig. 8k). Flow cytometry results showed that the tumor samples had the highest MB-MФ counts, with approximately 12% of the total macrophages in the tumor being MB-MФs, compared to the host macrophages (Fig. 8g). Additionally, MB-MФs comprised around 7% of the total collected leukocytes in the tumor samples (Fig. 8i), indicating substantial accumulation within the tumor microenvironment (TME). Viability analysis revealed that over 65% of the MB-MФs remained viable across all organs, regardless of the use of US imaging (Fig. 8j), suggesting that repeated US imaging did not significantly impact MB-MФ viability in the TME. Phenotype analysis showed no significant differences in polarization between the ultrasound and control groups (Fig. 8l). However, both groups exhibited a slight increase in M2-like phenotype markers within tumor samples, with higher expression of the M2 markers CD206 and Arg1. This M2-like increase is consistent with findings from other studies, where tumor-infiltrating macrophages (both BMDMs and RAW264.7) exhibited an increase in Arg1 expression^31^. In contrast, M1 markers such as CD86 and iNOS remained consistently low across all organ samples, indicating that pro-inflammatory activation was minimal in both groups. These findings suggest that US imaging did not lead to significant changes in macrophage polarization in the tumor microenvironment. Additionally, we observed a positive correlation between the number of mCherry-labeled MФs within the tumor, quantified via flow cytometry, and the US signal amplitude measured at the 8-hour timepoint in vivo (*p* = 0.018; Supplementary Fig. 14 in the manuscript). Although more comprehensive analysis is needed to draw definitive conclusions, these findings not only provide additional evidence that the US signals detected within tumors originated from MB-MФs but also suggest that the US signal can be used to infer the infiltration of MB-MФs into the tumor. In aggregate, these data demonstrated the ability of MB-MФs to travel through the vasculature, sense the tumor and penetrate the TME and provided evidence that this process can be tracked and quantified with US imaging.

## Discussion

In this study, we established methods and protocols to label MФs from mice and humans with MB ultrasound contrast agents and demonstrated that MB labeling and sonications at low mechanical index do not affect macrophage viability, migration capabilities, and phenotype (Figs. 1–3 and 8), supporting the potential of US imaging to nondestructively detect and track macrophage trafficking. Despite the significant damping exerted on the MB oscillations (Fig. 4), the MB displays highly nonlinear behavior upon sonication allowing to visualize MB-labeled macrophages with high specificity using nonlinear US imaging methods. Moreover, our combined in vitro and in vivo investigations revealed that the detection limit of MB-MФ approaches the theoretical limit of one MB-labeled macrophage (Figs. 5 and 7). These findings not only corroborate past in vitro investigations using MB-labeled neural progenitor cells^32^, but also indicate that this detection limit also holds true under in vivo conditions, allowing to track macrophage trafficking in solid tumors following intravenous delivery (Fig. 8). The observed detection limit is more than 2 orders magnitude lower compared to other clinical imaging modalities^13^ and comparable to dedicated miniPET systems^33^ but at significantly higher image resolution (≈100 µm). Together, these capabilities can potentially bridge the information gap that exists between optical methods and the 3D snapshots acquired with PET and MRI to support the discovery of new biomarkers for the diagnosis and prognosis of solid tumors.

Our investigations also revealed that the proposed approach could be used for image-guided cell injection procedures (Fig. 6 and Supplementary Fig. 9), where mis-injection of therapeutic cells can occur as high as 50% of the time in the absence of effective image guidance^13^. Importantly, these data were collected using conventional and readily available B-Mode imaging and FDA approved MBs, which significantly reduces the barrier for translation. Moreover, the conditions identified in our study and ability to observe the accumulation of the MB-MФs in the TME following intravenous injection may also support the development of effective image-guided cell therapy (e.g., CAR- MФs)^7^ and drug-release strategies (e.g., drug carrying MФs)^24^ by enabling new ways to track and interact with cell-based therapeutics.

Additionally, our analysis suggests that the phagocytosed MBs experience increased damping as compared to free bubbles, possibly due to the viscoelastic properties of the surrounding cellular compartments^34,35^. Hence, designing MBs with less damping and employing US frequencies closer to the resonance of the phagocytosed MB (e.g., 2–6 MHz) may further improve their effective scattering cross section, thereby allowing imaging with even higher sensitivity. The US imaging can also be further refined to accurately capture specific tissue segment by incorporating (non-rigid) registration strategies and super localization methods and techniques (i.e. super-resolution ultrasound)^36,37^ to overcome tradeoffs between penetration depth and resolution^38^. Effective fusion of these datasets with immunofluorescent imaging will provide a unique multiscale assessment of macrophage trafficking, whereby MФ trajectories, distribution, and interaction with the host cells can be combined to gain new insights into this complex biological process and inspire new diagnostic and therapeutic applications. Importantly, the ability to package MBs inside MФs may critically expand the diagnostic capabilities of ultrasound imaging by alleviating challenges of current MB ultrasound contrast agents associated with short circulation time (a few minutes)^39^ and inability to extravasate and engage with nonvascular diseased sites and cells.

Moreover, further refinements in labeling and MB properties may lead to unique MB responses and improved retention, potentially allowing US-based differentiation between M1 and M2 macrophage phenotypes. These studies can be further supported by investigations assessing the relationship between macrophage phenotype, microbubble properties and exposure conditions (US pulse amplitude, duration, repetition frequency, etc.) and their impact on macrophage phenotype and microbubble retention and echogenicity. Understanding and controlling the impact of macrophage polarization (M0 vs. M1 vs. M2 phenotypes^31^) on their labeling and trafficking characteristics can allow to further expand the proposed methods and concepts. Likewise, more targeted and in-depth future investigations to assess the potential of the proposed methods to differentiate between different tumor models (e.g., highly versus less aggressive^8^) and their response to treatment (e.g. chemotherapy^40^, CAR macrophages^7^, etc.) will allow to further establish the clinical implications of the proposed research and ability to extract prognostic markers. Moreover, extending our findings with BMDMs will allow to assess the proposed methods at a range of diseases, including atherosclerosis and cardiovascular diseases, where macrophages have shown similar promise for diagnosis and therapy^41,42^. Moving forward it will also be critical to understand the implications and clinical relevance of early, sustained, and late macrophage infiltration in the diseased site and use the appropriate imaging strategies.

While our current investigations were primarily focused on macrophages (from mice and humans) as they are particularly suited for labeling with MBs due to their innate phagocytic abilities, we have shown that other immune cells with similar phagocytic properties, such as dendritic cells (DCs)^43^, can be labeled (Fig. 2g) and potentially be tracked using the proposed methodology. Although the proposed approach does not require specific ligand, further investigations, possibly with targeted MBs, might allow to improve labeling efficacy and specificity as well as allow labeling specific cell sites (e.g. cell surface). The possibility of acoustic identification of macrophages and DCs or other cells is also intriguing, and it should be possible, especially if the rate of microbubble uptake and/or clearance by these cells are different. Further investigations in this direction, potentially using MBs with different properties (e.g., radius, composition, and rigidity)^44^ are warranted.

In summary, the proposed robust and clinically relevant MФ labeling protocol using FDA approved MBs, along with the effective imaging performance characteristics attained in the current investigations that employed exposures below the FDA safety levels (MI < 1.8) provide a noninvasive, portable, and scalable platform to study the complex problem of immune cell trafficking in solid tumors while creating new possibilities for the diagnosis and treatment monitoring of cancer that could also be deployed across a range of diseases^45^.

## Methods

### Cell culture

Raw 264.7 macrophages (MΦ) expressing mCherry (gift from Blazeck lab) were cultured in Dulbecco’s Modified Eagle Medium (DMEM; Thermo Fisher, 10566024) and were supplemented with 10% fetal bovine serum (FBS; Thermo Fisher, 10082147) and 1% penicillin/streptomycin at 37 °C in a humidified atmosphere of 5% CO_2_. 4T1-Fluc-Neo/eGFP-Puro cells expressing firefly luciferase were obtained from Imanis Life Sciences and were cultured in Roswell Park Memorial Institute (RPMI) 1640 medium and were supplemented with 10% fetal bovine serum (FBS) and 1% penicillin/streptomycin at 37 °C in a humidified atmosphere of 5% CO_2_. M1 phenotype was achieved by stimulating the RAW 264.7 cells with IFN-γ (100 ng/mL; Thermo Fischer, RP-8617) and LPS (20 ng/ml; Thermo Fischer, 00-4976-93) for 24 h. Untreated RAW 264.7 cells were considered as M0. All the experiments were conducted with RAW 264.7 macrophages with passage numbers between 18 and 25.

### Preparation of MB- MΦ

MΦs were cultured on tissue culture treated 35 mm dishes overnight (Nunc EasYDish Dishes; Thermo Fisher, 150464) or well plate (Falcon 96-Well, 353072) for optical characterization. MBs (Definity) were added the next day and dish/well plate was flipped in the incubator to allow for the buoyant microbubbles to come in contact with the adhered MΦ. Sterile parafilm (Parafilm, PM996) was used to seal the inverted dish. MΦ were incubated with the MBs for a range of different time points between 2–8 h. After incubation, the dish was washed 3 times with PBS to remove any free microbubbles. For the experiments where MB-MΦ were injected, after washing, gentle scraping was done and MB-MФs were harvested. Since this step could lead to some free bubbles, the harvested cells were further centrifugated at very low speed (100 × *g*) for 1 min to further separate them from free bubbles, while avoiding pellet formation which could lead to damage of the MB-MΦ during centrifugation.

### Microbubbles

The MBs used in the present study are listed in Supplementary Table 1. We prepared the in-house MBs according to established protocols^46,47^. The MB with a lipid only shell was prepared using a 9:1 molar ratio of 1,2-distearoyl-sn-glycero-3-phosphocholine (DSPC, 850365 C) and 1,2-distearoyl-sn-glycero-3-phosphoethanolamine-N-methoxy(polyethylene glycol)-2000 (DSPE-PEG2000, 880128) (Avanti Polar Lipids, Alabaster, AL, USA) in a solution containing propylene glycol 15% (v/v), glycerol 5% (v/v) and phosphate-buffered saline (PBS) 80% (v/v). After degassing of the lipid solution in 3-mL glass vials, the headspace of the vial was filled with either octafluoropropane gas (C3F8) or decafluorobutane gas (C4F10). The MBs with a mannose/lipid shell was prepared using a 9:0.5:0.5 molar ratio of 1,2-distearoyl-sn-glycero-3-phosphocholine (DSPC), 1,2-distearoyl-sn-glycero-3-phosphoethanolamine-N-methoxy (polyethylene glycol)-2000 (DSPE-PEG2000) (Avanti Polar Lipids, Alabaster, AL, USA) and 1,2-distearoyl-sn-glycero-3-phosphoethanolamine-N-(polyethylene glycol)-Mannose (Xi’an Ruixi Biotechnology, China, R-0221) in a solution containing propylene glycol 15% (v/v), glycerol 5% (v/v) and phosphate-buffered saline (PBS) 80% (v/v). After degassing of the lipid solution in 3-mL glass vials, the headspace of the vial was filled with either octafluoropropane gas or decafluorobutane gas. MBs were formed by agitation using a Vialmix device (Lantheus Medical Imaging, North Billerica, MA, USA).

### MΦ and MB-MΦ sonication

The setup included a well plate with mylar film on top and focused ultrasound transducer (FUS) along with a passive cavitation detector (PCD) sonicating and receiving echoes from the bottom. The FUS transducer is driven at 0.5 MHz with 20 cycles and increasing MI generated by a function generator (Picoscope 5000D, Pico Technology) and further amplified by a 50-dB power amplifier (model 240 L, Electronics & Innovation Ltd). The short number of cycles were chosen to prevent any streaming effects that can affect cell viability. On the receiver side, the PCD is connected to a high pass filter with a cut-off at 0.6 MHz to prevent saturation due to strong fundamental frequency signal. The signal is then fed to a data acquisition system and analyzed using fast Fourier transform from the host computer. Pressure of 0.5 MHz FUS transducer (Sonic Concept) used in this study were calibrated in water (i.e., free field), through 2 mm needle hydrophone (Precision Acoustics). Mechanical Index was calculated $\left(\mathrm{Mechanical}\,\mathrm{Index}=\frac{\mathrm{Peak}\,\mathrm{Negative}\,\mathrm{Pressure}\,[\mathrm{MPa}]}{\sqrt{\mathrm{Center}\,\mathrm{frequency}}[\mathrm{MHz}]}\right)$. The different spectral components were quantified as follows: (1) the harmonics were the average from 2f_0_ ± 5 bins, (2) the ultra-harmonics were average from 1.5f_0_ ± 5 bins, and (3) the broadband were at 5.72f_0_ ± 5 bins.

For sonication under high frame rate microscopy, we designed a setup on acrylic water tank which included a geometrically aligned FUS transducer (A305, 0.5 MHz center frequency, 1.25 inch focal distance; Olympus) at 30 degrees incident angle and placed at 2.5 inches from microfluidic channel (Ibidi µ-slide, channel height 200 µm). The designed setup was placed on a microscope with 60x objective lens (Nikon Ti2, CFI Plan Fluor 60XC, Nikon), which was connected to a high frame rate camera (HPV-X2, Shimadzu). The FUS transducer used in high frame rate microscopy was calibrated in water using 2 mm needle hydrophone (Precision Acoustics) at 2.5 inches, as used in our designed setup. When comparing the dynamics of MB and MB-MΦ, both schemes were sonicated with 0.21 MI and imaged at 5 MHz frame rate. The location of imaging was minimally changed for identical acoustical exposures to both schemes. To prevent the non-specific bindings of MBs to the channel walls, which can affect MB dynamics, before injecting MBs, the microfluidic channel in our setup was incubated with Bovine Serum Albumin (BSA) at room temperature for 1 h. MB dynamics video can be found in Supplementary Movie 2.

### Viability assay

Viability was assessed using ReadyProbes Cell Viability Imaging Kit (Life Technologies, Gaithersburg, MD, USA). The NucBlue Live reagent stains all nuclei, while NucGreen Dead reagent stains only dead cells. The percentage of dead cells was assessed by counting the number of dead cells to total number of nuclei.

### Bone marrow-derived macrophages

Femurs and tibias from 6–8 weeks old female BALB/c mice (000651, The Jackson Laboratory) were resected at day 0. Bone marrow from extracted limbs was flushed by ice-cold PBS into 50 mL conic tube until the bones were empty. After centrifuging the cells (5 mins, 200 × *g*, 4 °C) and removing PBS, red blood cells were lysed by incubating the marrow with 2 mL of lysing (ACK Lysing Buffer, VWR) for 3 min. Ice-cold complete medium (DMEM Glutamax + 10% FBS + 1% penicillin/streptomycin) was then added to neutralize the lysing agent, and the cells were again centrifuged (5 mins, 200 × *g*, 4 °C), the supernatant was removed, and differentiation medium was added (complete medium + 120 ng/mL M-CSF)(Gibco, PMC2044). Lastly, the solution was filtered through a 70-um strainer and seeded in 35 mm Petri dishes. Cells were kept in a humidified, 5% CO_2_ incubator at 37 °C until day 4. On day 4, 50% differentiation medium was added into each petri dish. On day 7, the supernatant was discarded, and the cells were incubated with 0.05% Trypsin for 3 min, adherent cells were then collected with a cell scraper for further studies.

### Human monocytes isolation

Peripheral blood was drawn from healthy human donors, as approved by the Georgia Institute of Technology Institutional Review Boards (IRB H22428). Healthy volunteers provided their informed consent for the collection, storage, and reuse of their samples. Standard phlebotomy techniques were used to obtain blood. Briefly, 60 mL of blood was drawn slowly through a 21 gauge needle into a 60-mL syringe containing 0.21 mL of a 1000-USP/mL solution of heparin sodium (Fisher Scientific) in PBS to achieve a heparin concentration of 3.5 USP/L in the collected blood. Complete blood counts were also performed on all donor samples. All isolation experiments were run within 4 h of the blood draw. Human CD14^+^ monocytes were isolated from freshly collected heparin-anticoagulated blood using the EasySep Direct Human Monocyte Isolation Kit (Stemcell Technologies, Vancouver, Canada, 19669) according to the manufacturer’s instructions. Briefly, 3 mM of EDTA was added to the collected blood, and a cocktail of biotinylated antibodies targeting non-desired cells such as granulocytes, T cells, B cells, NK cells, dendritic cells, platelets, and erythroid cells was added in combination with magnetic beads. The solution was then placed into EasySep magnet and incubated at room temperature. The enriched monocyte fraction was then collected and the selection process repeated 3 times. After the final round, the cells were counted and seeded in petri dish.

### Human macrophages and dendritic cells differentiation

Freshly seeded monocytes were seeded at a density of 0.5 × 10^6^ cells/mL in petri dishes in RPMI 1640 medium supplemented with 10% heat-inactivated FBS and 1% antibiotic-antimycotic. For macrophages, recombinant human macrophage colony-stimulating factors (M-CSF; Gibco, PHC9504) and IL-4 (PeproTech, 200-04-1MG) were added at a final concentration of 30 ng/mL and 25 ng/mL, respectively. For dendritic cells, recombinant human granulocyte-macrophage colony-stimulating factor (GM-CSF; Peprotech, 300-03-20UG) and IL-4 were added at a final concentration of 100 ng/mL and 25 ng/mL, respectively. Cells were cultured at 37 °C in a humidified 5% CO₂ incubator, with differentiation refreshed after the 5th day and then after every 2 days. After 7 days, morphological changes and adherence were monitored to confirm the differentiation of monocytes into macrophages/ dendritic cells.

### Cytokines analysis

BMDMs and RAW264.7 macrophages were seeded overnight at a density of 45k per well (96-well plate) in complete medium. The following day, we injected MB (5 uL per well) and filled up the well with complete medium, sealed them with parafilm, flipped the well plate and waited for 4 h. Control groups were processed in the same fashion except they were not labeled with MBs, M1 and M2 positive control were established by exposing seeded RAW264.7 macrophages (45k cells in 96 well plates) for 24 h to 200 ng/mL of LPS + 2.5 ng/mL of IFN-gamma and 10 ng/mL of IL-4 (Thermo Fischer, RMIL4I) + 10 ng/mL of IL-10 respectively. The cells were then rinsed 3X with PBS to remove excess MBs and incubated with 200 uL of media. After a 24 h incubation, the supernatant media was collected, centrifuged at 1500 g to remove the debris. The levels of the cytokines IL4, IL6, IL10, and TNF from MB-BMDMs, non-labeled BMDMs, MB-RAW 264.7, non-labeled RAW 264.7, and M1/M2 RAW264.7 positive controls were assessed by ELISA (arigoPLEX Mouse M1/M2 Cytokines multiplex ELISA Kit (IL4, IL6, IL10, TNF)). Human macrophages were processed in a similar fashion with ELISA (arigoPLEX Human M1/M2 Cytokines Multiplex ELISA Kit (IL4, IL6, IL10, TNF)).

### MΦ migration assay

Migration assay of MФs was performed using 24-well polycarbonated membrane insert with 3 μm pore size (Corning, CLS3415). In the bottom chamber, 2 × 10^5^ 4T1 cells were seeded in 6 wells overnight and RPMI were added to 6 other wells as control. MB-MΦ and MΦ were then seeded in the upper chamber at a density of 2 × 10^3^ cells. After 12 h of incubation, 20x fluorescent microscopy was done to assess the number of migrated MФ and MB-MФ. The same procedure was followed for BMDMs except that 4T1 presence was replaced by CCL2 gradient.

### Ultrasound imaging

Prior to imaging, MI for US transducer (Vermon, France) was determined using a calibrated hydrophone (Onda, Sunnyvale, CA, USA) with a reported uncertainty of ±10% in the frequency range of the excitation. The MI exhibited values within the range of 0.08 to 0.9, corresponding to Verasonics driving voltages spanning from 3 to 30 volts. MB-MΦ and MΦ (control) were injected into a hollow chamber in an acoustically transparent phantom made of 16% gelatin w/v. The hollow chamber had a diameter of 1.6 mm. Ultrasound images of this control volume was taken at various MI ranging from 0.08–0.8 with two imaging sequences, both using a L22-14V transducer (Vermon, France) connected to a programmable ultrasound scanner (Verasonics Vantage). Firstly, B-Mode at 15.625 MHz, pulse width of 0.67, 4 half cycles, positive starting polarity were parameters used to configure the transmit/excitation waveform. Secondly, non-linear imaging in the form of Amplitude Modulated Pulse Inversion at 15.625 MHz where three pulses are transmitted—One pulse at full amplitude and negative polarity, one pulse using even elements only and positive polarity and one pulse using odd elements only and positive polarity^39,48,49^. Normalized Intensity and contrast with respect to the background were analyzed. We used the following expressions to estimate the axial and lateral resolution of the imaging system: ${\mathrm{Lateral}}=1.44\frac{\lambda z}{2\alpha }=40.5\,\mu {\rm{m}}$, and $\mathrm{Axial}=\frac{\lambda \times \mathrm{num}.\mathrm{cycles}}{2}=98.5\,\mu {\rm{m}}$, with

$$\left. \lambda=98.5\,{{\mu }}{{m}},\,2\alpha=28\,{mm},\,z=8\,{mm},\,c=1540\frac{{{m}}}{{{s}}}\right]$$

### In vitro experiments using the gelatin phantom

To evaluate the detection limit in vitro, the imaging volume was calculated and imaged with progressively reducing concentration, starting from ${3.11\times 10}^{5}$ cells/ml and progressing to ${3.11\times 10}^{2}$ cells/ml with 10-fold dilutions. Considering that the sonication volume was approximately 3.2 µl, the anticipated cell count within this volume was estimated to be from 1000 cells to 1 cell.

### In vivo tumor model

All animal procedures were performed according to the guidelines of the Public Health Policy on the Humane Care and Use of Laboratory Animals and approved by the Institutional Animal Care and Use Committee of Georgia Institute of Technology. Animals were maintained in a specific pathogen-free (SPF) barrier facility, with ad libitum access to standard chow and water, and housed under a 12-h light/dark cycle at a controlled ambient temperature (72 °F) and humidity (30–70%). Experimental and control animals were co-housed where appropriate. For all procedures requiring tissue collection, animals were anesthetized with 5% isoflurane and subsequently euthanized by cervical dislocation while under deep anesthesia, in accordance with approved humane endpoints. All in vivo experiments were performed on 8–12 weeks female Balb/C mice (000651, The Jackson Laboratory) under protocols approved by the Institutional Animal Care and Use Committee at the Georgia Institute of Technology. To establish a breast tumor model, 2 × 10^6^ 4T1 cells were injected into the posterior upper flank of each mouse on each side and were allowed to grow till they reached a size of 1.5–2 mm which usually took about 3 weeks from inoculation. The upper flank was chosen to reduce motion artifacts that may arise from breathing. All mice received lymphodepletion 18–20 h prior to macrophage and MB-macrophage injection with a combination of cyclophosphamide (3 mg/mouse) and fludarabine (1 mg/mouse) administered through intraperitoneal injection (i.p.). More specifically, cyclophosphamide (Cayman Chemicals, NDC 10019-955-01) was resuspended in sterile saline at 40 mg/ml immediately prior to use, and 3 mg per mouse was administered i.p. Fludarabine phosphate (Cayman Chemicals, NDC 25021-242-02, 25 mg/ml) was stored at 4 °C, and 1 mg per mouse was administered Intraperitoneally. Tumor growth was monitored by caliper measurements every 5 days. The maximum tumor burden permitted by the Institutional Animal Care and Use Committee protocol was a tumor diameter of 1.5 cm in any dimension. All animals were monitored to ensure they remained within this limit. Pre-defined endpoint criteria for early euthanasia to minimize animal distress included reaching the maximum tumor size, weight loss greater than 10%, or the presence of other signs of distress such as hunched posture, reduced activity, or labored breathing. No animals were terminated early due to exceeding these humane endpoints during the imaging study.

### Isolation of leukocytes in tissue sample and flow cytometry

To evaluate the population and viability of systemically delivered MB-MФs we performed flow cytometry on harvested tissue samples. After the 8-hour imaging time point, mice were euthanized, and both the tumor and other organs, including spleen, lung, and liver were collected. The harvested tissues were immediately minced using sterile scalpels. The tissue fragments were then washed with PBS and enzymatically digested with a cocktail of collagenase IV (Worthington, 3.2 mg/mL) and deoxyribonuclease I (Worthington, 1 mg/mL) diluted in sterile PBS. Following digestion, the cells were filtered through a 70 μm cell strainer, washed with sterile PBS, and suspended in 70% Percoll (Cytiva) solution. The suspensions were overlaid with 30% Percoll and HBSS, and subsequently centrifuged for 30 min at 650 × *g*. Enriched leukocyte populations were recovered at the 70% to 30% Percoll interface and washed with 1XPBS.

The flow cytometry staining for both in vivo and in vitro samples was performed as follows: single cell suspensions (0.5–1 × 10^6^ cells) from either leukocytes-enriched tissue samples or in vitro differentiated cultures were incubated with viability dye (Zombie Aqua, Biolegend) at room temperature for 15 min. Samples were then washed with excess buffer and resuspended in FC buffer (1X PBS, 0.5% FBS, 2 mM EDTA). To reduce non-specific binding, cells were incubated with Fc block for 15 mins prior to antibody staining. The following antibodies were used for surface and, where applicable, intracellular staining (see Supplementary Table 2 for more details): F4/80 (PerCP-Cy5.5 or BV650; Biolegend), CD11b (PE-Cy7 or PerCP-Cy5.5; Biolegend), CD86 (BV785 or BV421; Biolegend), iNOS APC (Biolegend), CD206 (Alexa700 or PE-Cy7; Biolegend), and ARG1 PE (Biolegend). These markers were selected to assess RAW 264.7 cells and BMDMs. For in vitro differentiated human macrophages and dendritic cells, human-specific antibodies were used, including CD14 Alexa488, CD11b PerCp-Cy5.5, CD68 APC, CD80 PE, CD163 APC-Cy7, CD1a PE, and CD83 PE-Cy7 (all Biolegend). Cells were fixed between surface and intracellular staining steps, and all samples were analyzed using a Cytek Aurora flow cytometer (Cytek). Gating strategies and representative plots are shown in (Supplementary Figs. 1, 2 and 13).

### Ultrasound imaging in vivo

Mice were maintained under 1.5% isoflurane anesthesia for the duration of the experiment on a temperature-controlled heat pad. Following depilation (Nair) over tumor area, a 25-g needle was inserted into the tumor. L22-14v transducer connected to a programmable ultrasound scanner (Verasonics Vantage 256) was then fixed to the plane where the needle intensity was maximum. 200 µl of MΦ/ MB-MΦ were then injected into the tumor via infusion at the rate of 100 μl/min for 2 min with a concentration of 1 × 10^7^ MB-MΦ/ml for the data sown in Fig. 6b and 1 × 10^6^ MB-MΦ/ml for the data shown in Fig. 6e. To evaluate the detection limit in vivo, we imaged progressively reducing concentration, starting from ${10}^{5}$ cells/ml and progressing to 10 cells/ml with 10-fold dilutions. We infused 200 µl of MB-MΦ resulting in cell counts at the tip of the needle ranges from 2x${10}^{4}$ cells to less than 2 cells. For the intravenous injection 200 µl of MB-MΦ were injected intravenously via tail vein injection at the rate of 100 μl/min for 2 min with a concentration of 1 × 10^7^ MB-MΦ/ml, resulting in 2x${10}^{6}$ cells.

### Vascular mapping with MB-enhanced US and US image registration

Following anesthesia, the L22-14v transducer was positioned using a manual 3D positioning system at the distal end of the tumor, starting from the side proximate to the abdomen. The placement was guided using the tissue only images of each plane (approximately 0.75 mm apart) of the tumor acquired during vascular imaging. A total of 5.5 × 10^7^ microbubbles were administered into the tail vein of the anesthetized mice with repeated bolus injection after every 10 min, followed by image acquisition of each plane initiated from the distal end of the tumor. Contrast enhanced US images were acquired using the Amplitude-Modulated Pulse Inversion method with 128 ray lines (focus transmit) and 128 receive acquisitions at 15.625 MHz transmit frequency and augmented using super resolution imaging framework. In each plane, 700 frames of receive data were captured and the beamformed image data was stored in matrix format. MB detection was augmented by applying Singular Value Decomposition (SVD) to the Casorati matrix of the stack of 700 images^50^. The initial 10% of singular values are indicative of tissue clutter and the final 15% associated with noise were truncated to 0. The singular value thresholds were determined^51^. Post-SVD filtering, Richardson-Lucy deconvolution was employed with a position independent uniform Gaussian approximation of the point spread function to further isolate the echoes from multiple microbubbles that are closer than a few wavelengths in each frame^51^. Finally, the maximum intensity projection of the vasculature was displayed after interpolation and smoothing to obtain the final vascular images in each plane^52^. The register vascular images, which were collected one day before the experiments with MB-MФ administration, the contrast enhanced US image stack was filtered using only the first 10% of singular values (representative of tissue clutter) and truncating the remaining singular values to 0^51,53^. These tissue-only images served as a reference for visual template matching during and immediately after MB-MФ administration as well as 4- and 8-h.

### Immunofluorescence staining and microscopy

Tissues were first prepared for staining by fixing in 4% paraformaldehyde at room temperature for 10 min. After washing with phosphate-buffered saline (PBS), the sections were first blocked for 1 h at room temperature (2% bovine serum albumin and 5% goat serum in PBS) and then incubated with primary antibody diluted in 1% bovine serum albumin (1:100) for 12 h at 4 °C. Rabbit anti-mouse CD31 (ab28364, Abcam Inc.) was used for vessel staining. Next, the sections were incubated with goat anti-rabbit Alexa Fluor 647 secondary antibody diluted in 1% bovine serum albumin (1:250; A31556, Invitrogen) for 1 h at room temperature. To stain the cell nucleus, samples were incubated with DAPI diluted in PBS (1:1000; 62248, Invitrogen) for 10 min after washing. Last, the sections were rinsed with PBS to remove excess antibody, mounted with mounting medium (Prolong Glass Antifade Mountant, Invitrogen), and covered with coverslips. Samples were cured with a mounting medium for 24 h in the dark at room temperature before imaging.

Cells were fixed with 4% paraformaldehyde at 37 °C for 15 min and then permeabilized with 0.1% of Triton X-100 (0.1%) for 15 min at room temperature (only for iNOS). Next, the cells were incubated with 2% BSA in 1X PBS for 60 min to block non-specific binding of antibodies. The cells were then incubated with CD206, CD86 and iNOS antibodies for 3 h at room temperature. Between each step, cells were washed 3 times with PBS except for the last step during which cells were washed with 1X PBS-T.

### Statistical analysis

Data are presented as mean ± SD. Multiple groups were analyzed using one-way ANOVA or two-way ANOVA followed by post-hoc analysis using Tukey’s multiple comparison. Source data are provided with this paper. All statistical analyses were performed using GraphPad Prism 10.0.2. *p* values *p* ≤ 0.05 were considered statistically significant (n.s. no significance, *p ≤ 0.05, **p ≤ 0.01, ****p ≤ 0.0001).

### Reporting summary

Further information on research design is available in the Nature Portfolio Reporting Summary linked to this article.

## Supplementary information

Supplementary Information

Description of Additional Supplementary Files

Suppl. Movie 1

Suppl. Movie 2

Suppl. Movie 3

Reporting Summary

## Source data

Source Data

## Data Availability

All data are included in the Supplementary Information or available from the authors, as are unique reagents used in this article. The raw numbers for charts and graphs are available in the Source Data file whenever possible. The FACS data generated in this study have been deposited in the Zenodo database (10.5281/zenodo.15653071). Any additional requests for information can be directed to, and will be fulfilled by, the corresponding authors. Source data are provided with this paper.

## References

[1] Cassetta, L. & Pollard, J. W. Targeting macrophages: therapeutic approaches in cancer. *Nat. Rev. Drug Discov.* **17**, 887–904 (2018). 30361552 10.1038/nrd.2018.169

[2] Bruni, D., Angell, H. K. & Galon, J. The immune contexture and immunoscore in cancer prognosis and therapeutic efficacy. *Nat. Rev. Cancer* **20**, 662–680 (2020). 32753728 10.1038/s41568-020-0285-7

[3] Xia, W., Singh, N., Goel, S. & Shi, S. Molecular imaging of innate immunity and immunotherapy. *Adv. Drug Deliv. Rev.* **198**, 114865 (2023). 37182699 10.1016/j.addr.2023.114865

[4] Cassetta, L. et al. Human tumor-associated macrophage and monocyte transcriptional landscapes reveal cancer-specific reprogramming, biomarkers, and therapeutic targets. *Cancer Cell* **35**, 588–602.e10 (2019). 30930117 10.1016/j.ccell.2019.02.009 PMC6472943

[5] Binnewies, M. et al. Understanding the tumor immune microenvironment (TIME) for effective therapy. *Nat. Med.* **24**, 541–550 (2018). 29686425 10.1038/s41591-018-0014-x PMC5998822

[6] Luca, B. A. et al. Atlas of clinically distinct cell states and ecosystems across human solid tumors. *Cell* **184**, 5482–5496.e28 (2021). 34597583 10.1016/j.cell.2021.09.014 PMC8526411

[7] Klichinsky, M. et al. Human chimeric antigen receptor macrophages for cancer immunotherapy. *Nat. Biotechnol.* **38**, 947–953 (2020). 32361713 10.1038/s41587-020-0462-y PMC7883632

[8] Mantovani, A., Allavena, P., Marchesi, F. & Garlanda, C. Macrophages as tools and targets in cancer therapy. *Nat. Rev. Drug Discov.* **21**, 799–820 (2022). 35974096 10.1038/s41573-022-00520-5 PMC9380983

[9] DeNardo, D. G. & Ruffell, B. Macrophages as regulators of tumour immunity and immunotherapy. *Nat. Rev. Immunol.* **19**, 369–382 (2019). 30718830 10.1038/s41577-019-0127-6 PMC7339861

[10] Ritchie, D. et al. In vivo tracking of macrophage activated killer cells to sites of metastatic ovarian carcinoma. *Cancer Immunol. Immunother.* **56**, 155–163 (2007). 16733671 10.1007/s00262-006-0181-3 PMC11030026

[11] Entenberg, D., Oktay, M. H. & Condeelis, J. S. Intravital imaging to study cancer progression and metastasis. *Nat. Rev. Cancer* **23**, 25–42 (2023). 36385560 10.1038/s41568-022-00527-5 PMC9912378

[12] Miller, M. A. et al. Radiation therapy primes tumors for nanotherapeutic delivery via macrophage-mediated vascular bursts. *Sci. Transl. Med.* **9**, eaal0225 (2017). 28566423 10.1126/scitranslmed.aal0225 PMC6681815

[13] Bulte, J. W. M. & Daldrup-Link, H. E. Clinical tracking of cell transfer and cell transplantation: trials and tribulations. *Radiology* **289**, 604–615 (2018). 30299232 10.1148/radiol.2018180449 PMC6276076

[14] Weissleder, R., Nahrendorf, M. & Pittet, M. J. Imaging macrophages with nanoparticles. *Nat. Mater.* **13**, 125–138 (2014). 24452356 10.1038/nmat3780

[15] Ahrens, E. T. & Bulte, J. W. M. Tracking immune cells in vivo using magnetic resonance imaging. *Nat. Rev. Immunol.* **13**, 755–763 (2013). 24013185 10.1038/nri3531 PMC3886235

[16] Sehl, O. C. & Foster, P. J. The sensitivity of magnetic particle imaging and fluorine−19 magnetic resonance imaging for cell tracking. *Sci. Rep.* **11**, 22198 (2021). 34772991 10.1038/s41598-021-01642-3 PMC8589965

[17] Jeong, H. J. et al. Macrophage cell tracking PET imaging using mesoporous silica nanoparticles via in vivo bioorthogonal F-18 labeling. *Biomaterials* **199**, 32–39 (2019). 30735894 10.1016/j.biomaterials.2019.01.043

[18] Li, X., Rosenkrans, Z. T., Wang, J. & Cai, W. PET imaging of macrophages in cardiovascular diseases. *Am. J. Transl. Res.* **12**, 1491–1514 (2020). 32509158 PMC7270023

[19] Wilk, B. et al. Hybrid PET/MR imaging in myocardial inflammation post-myocardial infarction. *J. Nucl. Cardiol.* **27**, 2083–2099 (2020). 31797321 10.1007/s12350-019-01973-9 PMC7391987

[20] Kim, J. et al. Use of nanoparticle contrast agents for cell tracking with computed tomography. *Bioconjug. Chem.* **28**, 1581–1597 (2017). 28485976 10.1021/acs.bioconjchem.7b00194 PMC5481820

[21] Heinzmann, K., Carter, L. M., Lewis, J. S. & Aboagye, E. O. Multiplexed imaging for diagnosis and therapy. *Nat. Biomed. Eng.* **1**, 697 (2017). 31015673 10.1038/s41551-017-0131-8

[22] Thomas, S. *Diagnostic Ultrasound Imaging: Inside Out* 2nd edn (Academic Press, 2013).

[23] Hartono, D. et al. On-chip measurements of cell compressibility via acoustic radiation. *Lab Chip* **11**, 4072 (2011). 22020269 10.1039/c1lc20687g

[24] Xu, Z., Liu, H., Tian, H. & Yan, F. Real-time imaging tracking of engineered macrophages as ultrasound-triggered cell bombs for cancer treatment. *Adv. Funct. Mater.* **30**, 1910304 (2020).

[25] Kim, I. et al. Real-time, in situ imaging of macrophages via phase-change peptide nanoemulsions. *Small* **19**, 2301673 (2023). 10.1002/smll.202301673 PMC10787802 37452514

[26] Emanuel, A. L. et al. Contrast-enhanced ultrasound for quantification of tissue perfusion in humans. *Microcirculation* **27**, e12588 (2020). 31465606 10.1111/micc.12588 PMC7050534

[27] Hyvelin, J.-M. et al. Characteristics and echogenicity of clinical ultrasound contrast agents: an in vitro and in vivo comparison study. *J. Ultrasound Med.* **36**, 941–953 (2017). 28240842 10.7863/ultra.16.04059

[28] Tu, J. et al. Microbubble sizing and shell characterization using flow cytometry. *IEEE Trans. Ultrason. Ferroelectr. Freq. Control* **58**, 955–963 (2011). 21622051 10.1109/TUFFC.2011.1896 PMC4495763

[29] Li, P. et al. Comparative proteomic analysis of polarized human THP-1 and mouse RAW264.7 macrophages. *Front. Immunol.* **12**, 700009 (2021). 10.3389/fimmu.2021.700009 PMC8276023 34267761

[30] Eckersley, R. J., Chin, C. T. & Burns, P. N. Optimising phase and amplitude modulation schemes for imaging microbubble contrast agents at low acoustic power. *Ultrasound Med. Biol.* **31**, 213–219 (2005). 15708461 10.1016/j.ultrasmedbio.2004.10.004

[31] Aalipour, A. et al. Engineered immune cells as highly sensitive cancer diagnostics. *Nat. Biotechnol.* **37**, 531–539 (2019). 30886438 10.1038/s41587-019-0064-8 PMC7295609

[32] Cui, W. et al. Neural progenitor cells labeling with microbubble contrast agent for ultrasound imaging in vivo. *Biomaterials* **34**, 4926–4935 (2013). 23578557 10.1016/j.biomaterials.2013.03.020 PMC3742341

[33] Jung, K. O. et al. Whole-body tracking of single cells via positron emission tomography. *Nat. Biomed. Eng.* **4**, 835–844 (2020). 32541917 10.1038/s41551-020-0570-5 PMC7423763

[34] Lindner, J. R. et al. Noninvasive ultrasound imaging of inflammation using microbubbles targeted to activated leukocytes. *Circulation* **102**, 2745–2750 (2000). 11094042 10.1161/01.cir.102.22.2745

[35] Dayton, P. A. et al. Optical and acoustical dynamics of microbubble contrast agents inside neutrophils. *Biophys. J.* **80**, 1547–1556 (2001). 11222315 10.1016/S0006-3495(01)76127-9 PMC1301346

[36] Demené, C. et al. Transcranial ultrafast ultrasound localization microscopy of brain vasculature in patients. *Nat. Biomed. Eng.* **5**, 219–228 (2021). 33723412 10.1038/s41551-021-00697-x PMC7610356

[37] Heiles, B. et al. Performance benchmarking of microbubble-localization algorithms for ultrasound localization microscopy. *Nat. Biomed. Eng.* 1–12 10.1038/s41551-021-00824-8 (2022). 10.1038/s41551-021-00824-8 35177778

[38] Christensen-Jeffries, K. et al. Super-resolution ultrasound imaging. *Ultrasound Med. Biol.* **46**, 865–891 (2020). 31973952 10.1016/j.ultrasmedbio.2019.11.013 PMC8388823

[39] Averkiou, M. A., Bruce, M. F., Powers, J. E., Sheeran, P. S. & Burns, P. N. Imaging methods for ultrasound contrast agents. *Ultrasound Med. Biol.* **46**, 498–517 (2020). 31813583 10.1016/j.ultrasmedbio.2019.11.004

[40] Karagiannis, G. S. et al. Neoadjuvant chemotherapy induces breast cancer metastasis through a TMEM-mediated mechanism. *Sci. Transl. Med.* **9**, eaan0026 (2017). 28679654 10.1126/scitranslmed.aan0026 PMC5592784

[41] Moore, K. J., Sheedy, F. J. & Fisher, E. A. Macrophages in atherosclerosis: a dynamic balance. *Nat. Rev. Immunol.* **13**, 709–721 (2013). 23995626 10.1038/nri3520 PMC4357520

[42] Dick, S. A. et al. Self-renewing resident cardiac macrophages limit adverse remodeling following myocardial infarction. *Nat. Immunol.* **20**, 29–39 (2019). 30538339 10.1038/s41590-018-0272-2 PMC6565365

[43] Savina, A. & Amigorena, S. Phagocytosis and antigen presentation in dendritic cells. *Immunol. Rev.* **219**, 143–156 (2007). 17850487 10.1111/j.1600-065X.2007.00552.x

[44] Li, H. et al. Biomechanics of phagocytosis of red blood cells by macrophages in the human spleen. *Proc. Natl. Acad. Sci. USA* **121**, e2414437121 (2024). 39453740 10.1073/pnas.2414437121 PMC11536160

[45] Herron, T. & Gossman, W. *StatPearls* (StatPearls Publishing, 2024).

[46] Lindsey, B. D. et al. High resolution ultrasound superharmonic perfusion imaging: in vivo feasibility and quantification of dynamic contrast-enhanced acoustic angiography. *Ann. Biomed. Eng.* **45**, 939–948 (2017). 27832421 10.1007/s10439-016-1753-9 PMC5682933

[47] Shelton, S. E., Lindsey, B. D., Tsuruta, J. K., Foster, F. S. & Dayton, P. A. Molecular acoustic angiography: a new technique for high resolution superharmonic ultrasound molecular imaging. *Ultrasound Med. Biol.* **42**, 769 (2015). 26678155 10.1016/j.ultrasmedbio.2015.10.015 PMC5653972

[48] Shen, C.-C., Chou, Y.-H. & Li, P.-C. Pulse inversion techniques in ultrasonic nonlinear imaging. *J. Med. Ultrasound* **13**, 3–17 (2005).

[49] Brown, K. G. & Hoyt, K. Evaluation of nonlinear contrast pulse sequencing for use in super-resolution ultrasound imaging. *IEEE Trans. Ultrason. Ferroelectr. Freq. Control* **68**, 3347–3361 (2021). 34181537 10.1109/TUFFC.2021.3092172 PMC8588781

[50] Schoen, S. et al. Morphological reconstruction improves microvessel mapping in super-resolution ultrasound. *IEEE Trans. Ultrason. Ferroelectr. Freq. Control* **68**, 2141–2149 (2021). 33544672 10.1109/TUFFC.2021.3057540 PMC8574223

[51] Demené, C. et al. Spatiotemporal clutter filtering of ultrafast ultrasound data highly increases Doppler and fultrasound sensitivity. *IEEE Trans. Med. Imaging* **34**, 2271–2285 (2015). 25955583 10.1109/TMI.2015.2428634

[52] Siepmann, M., Schmitz, G., Bzyl, J., Palmowski, M. & Kiessling, F. Imaging tumor vascularity by tracing single microbubbles. in *2011 IEEE International Ultrasonics Symposium* 1906–1909 (IEEE, 2011).

[53] Desailly, Y. et al. Contrast enhanced ultrasound by real-time spatiotemporal filtering of ultrafast images. *Phys. Med. Biol.* **62**, 31 (2016). 27973352 10.1088/1361-6560/62/1/31

