# Supplementary material for: Imaging of macrophage accumulation in solid tumors with ultrasound
Source: Nat Commun. 2025 Jul 9;16:6322. doi: 10.1038/s41467-025-61624-1 (PMC12241657; doi:10.1038/s41467-025-61624-1)
Supplement: Supplementary file 1 — Supplementary Information [file 41467_2025_61624_MOESM1_ESM.pdf]

Imaging of macrophage accumulation in solid tumors with ultrasound

Ashley Alva [1*], Chulyong Kim [2*], Pranav Premdas [1*], Yann Ferry [3*], Hohyun Lee [2], Nidhi Lal [3], Jing Bowen [3], Edward Botchwey [3], Brooks Lindsey [3], Costas Arvanitis [2, 3, §]

[1]Electrical and Computer Engineering, Georgia Institute of Technology, Atlanta, Georgia, United States

[2]Woodruf School of Mechanical Engineering, Georgia Institute of Technology, Atlanta, Georgia, United States

[3]Coultier Department of Biomedical Engineering, Georgia Institute of Technology and Emory University, Atlanta, Georgia, United States

*****Equally contributing authors

[§] Corresponding author

Supplementary Information

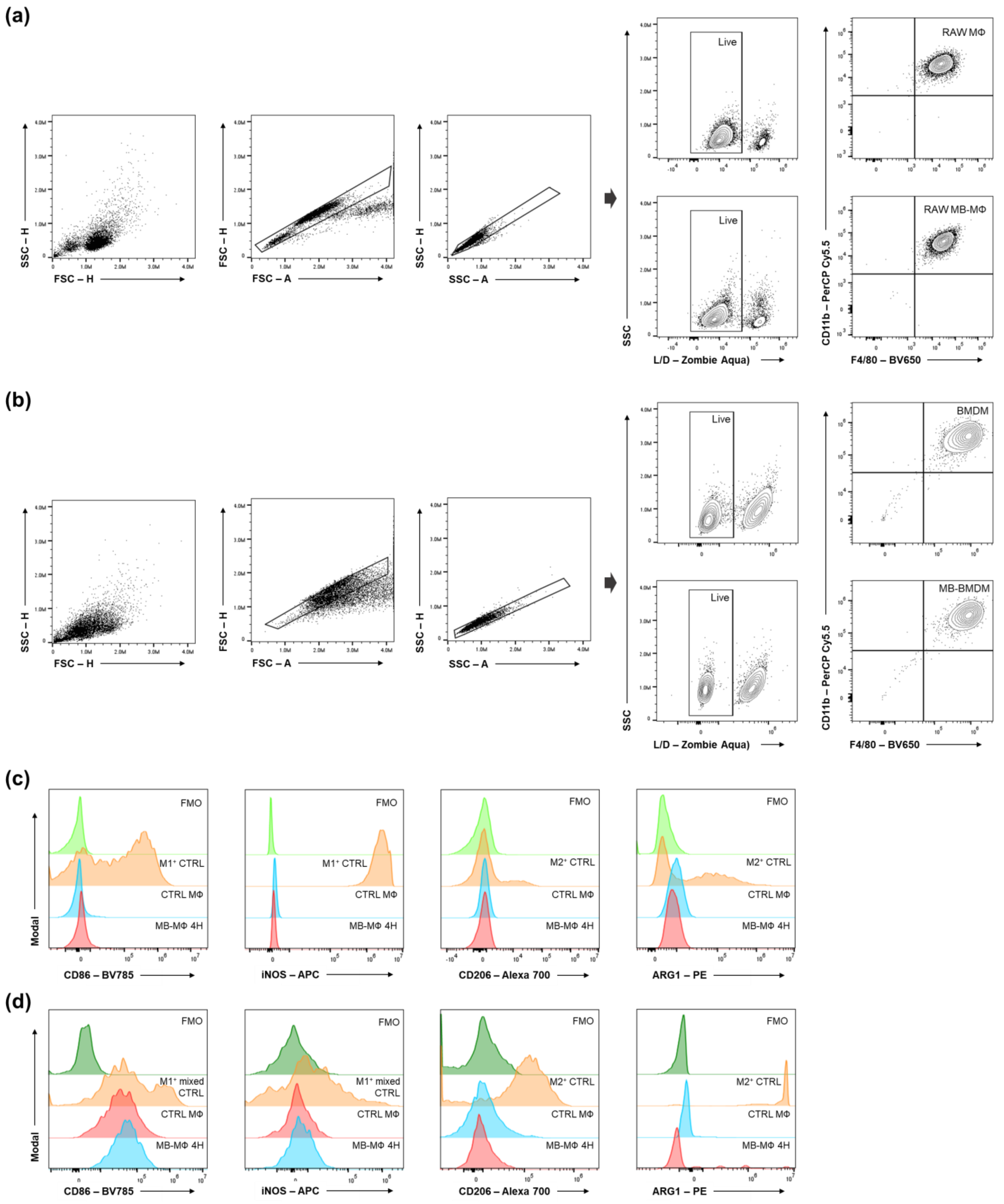

Supplementary Figure 1. Representative plots obtained from flow cytometry for RAW 264.7 and BMDM cells (in vitro). (a) Gating strategies for flow analysis of MΦ (top) vs. MB-MΦ (bottom) on RAW 246.7 cells. **(b)** Gating strategies for flow analysis of MΦ (top) vs. MB-MΦ (bottom) on bone-marrow derived macrophages (BMDMs). **(c-d)** Representative Fluorescence Minus One (FMO) controls for macrophage phenotype markers (CD86, iNOS, CD206, and ARG1) on RAW 246.7 cells (top) and BMDM cells (bottom).

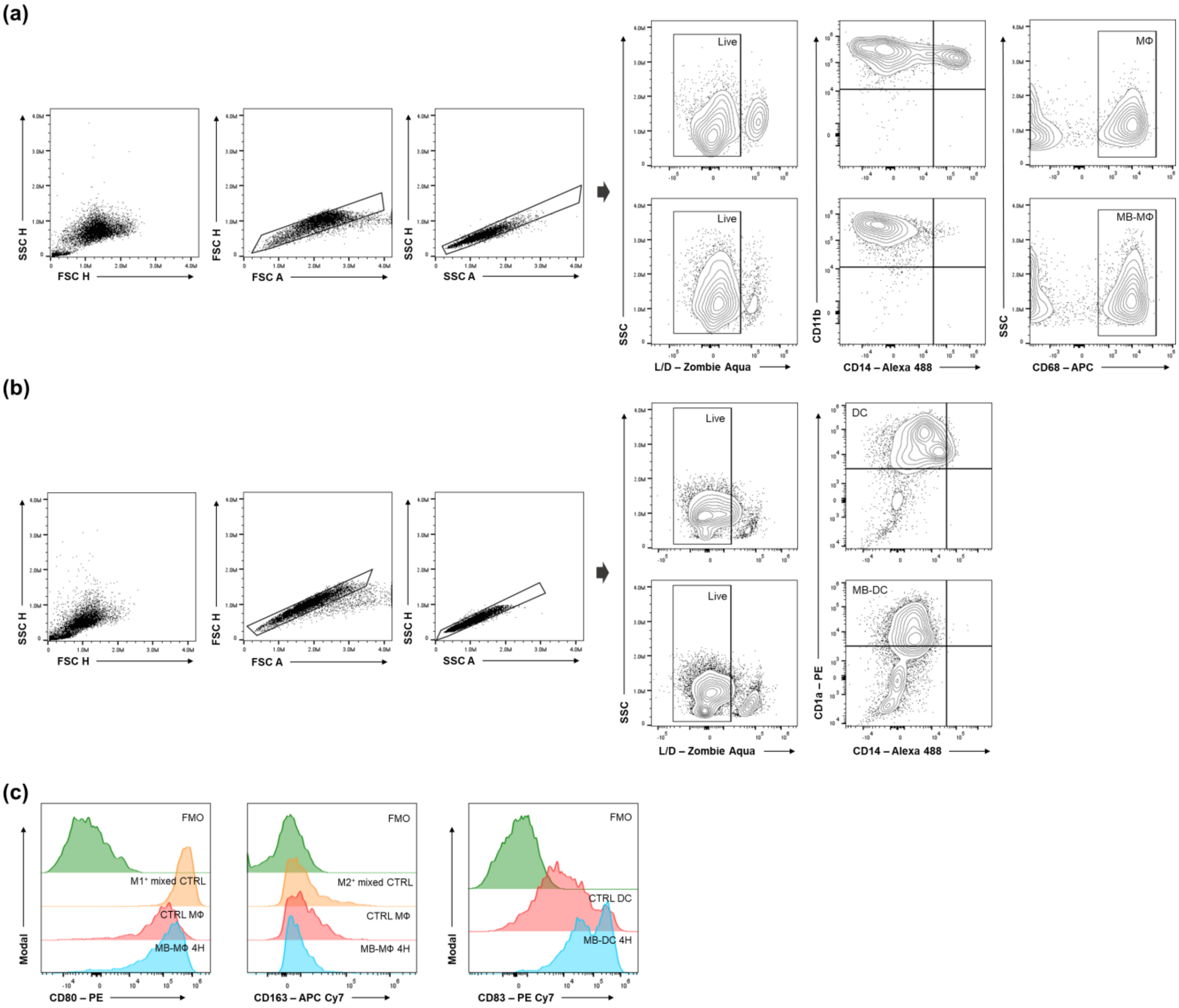

Supplementary Figure 2. Representative plots obtained from flow cytometry for primary human macrophages and dendritic cells (in vitro). (a-b) Gating strategies for flow analysis of Ctrl (top) vs. MB-labeled (bottom) on **(a)** primary human macrophages and **(b)** dendritic cells. **(c)** Representative Fluorescence Minus One (FMO) controls for surface markers (CD80, CD163, and CD83) on MB-labeled human cells.

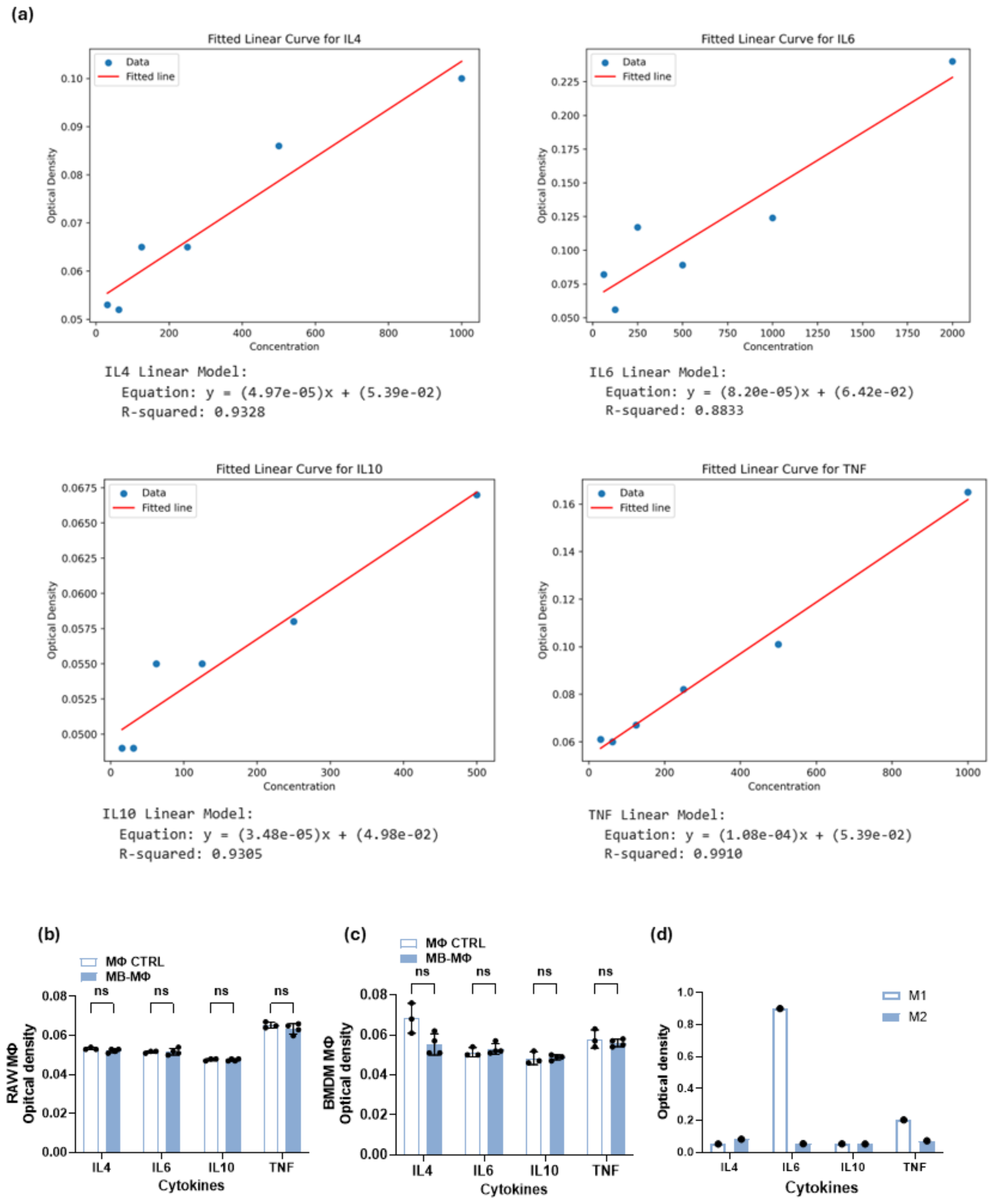

Supplementary Figure 3. Cytokine measurement RAW and BMDMs. (a) Standard curve for IL4, IL16, IL10, TNF **(b-c)** Optical density read by ELISA assay of IL4, IL6, IL 10, and TNF released in the 24 hours following RAW264.7 macrophages and BMDMs labeling compared to non-labeled. (n = 3 wells). Data are presented as mean values +/- SD. P values were determined by using a two-tailed unpaired t-test with Holm-Sidak correction; n.s. - not significant. **(d)** Positive control with polarized cells (M1: 200 ng/mL LPS + 2.5 ng/mL IFN-Gamma; M2: 10 ng/mL of IL4 +10 ng/mL of IL10; both incubated for 24 hours). Right after polarization, the media with cytokines was discarded, the cells were washed 3x with PBS, CDMEM was added, and the supernatant was collected 24 hours later.

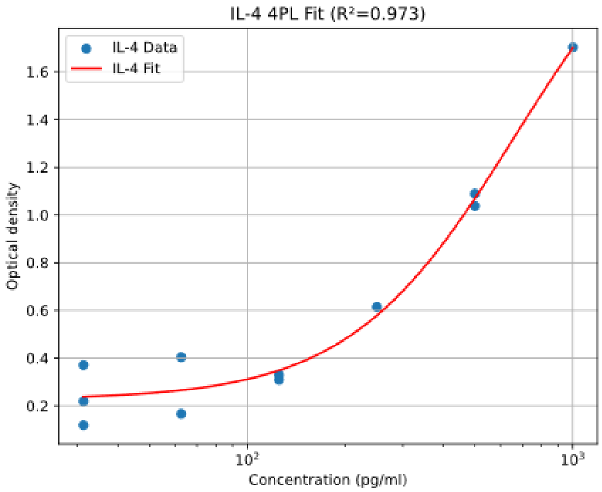


```
IL-4 4PL Model Parameters:
  A (Top asymptote):      2.441e+00
  B (Hill slope):         -1.697e+00
  C (Inflection point):   6.660e+02
  D (Bottom asymptote):   2.261e-01
  R²:                     0.9732
```

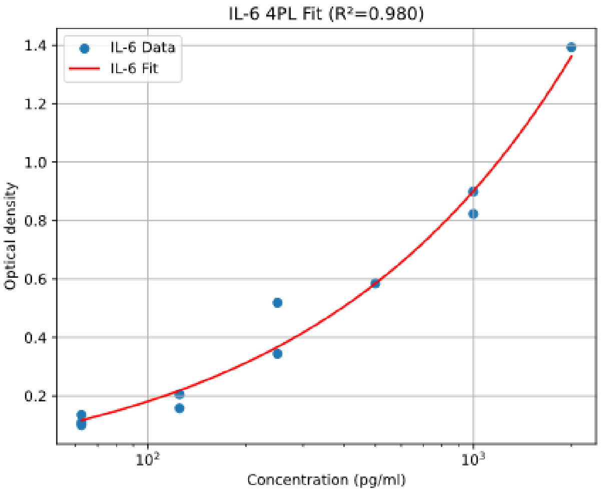


```
IL-6 4PL Model Parameters:
  A (Top asymptote):      6.608e+04
  B (Hill slope):         -5.431e-01
  C (Inflection point):   7.369e+11
  D (Bottom asymptote):   -1.075e-01
  R²:                     0.9798
```

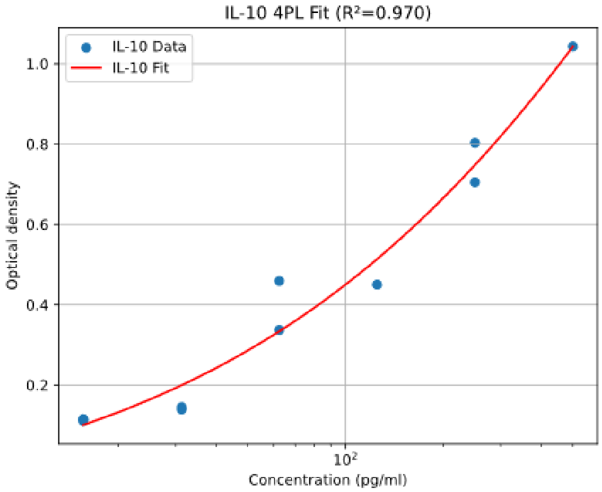


```
IL-10 4PL Model Parameters:
  A (Top asymptote):      4.781e+00
  B (Hill slope):         -5.221e-01
  C (Inflection point):   4.511e+03
  D (Bottom asymptote):   -1.426e-01
  R²:                     0.9700
```

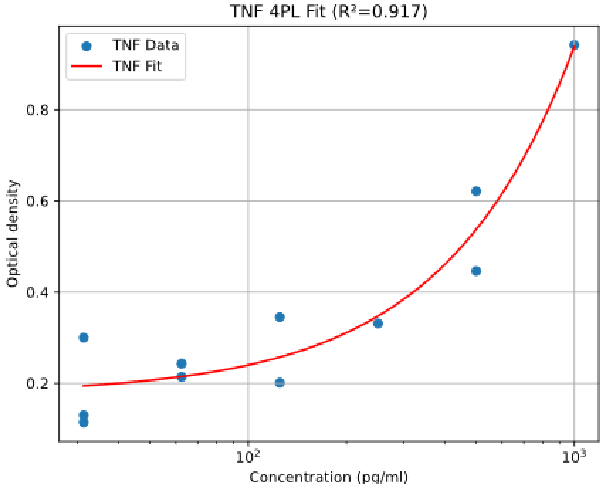


```
TNF 4PL Model Parameters:
  A (Top asymptote):      4.731e+04
  B (Hill slope):         -1.077e+00
  C (Inflection point):   2.821e+07
  D (Bottom asymptote):   1.760e-01
  R²:                     0.9172
```

Supplementary Figure 4. Cytokine measurement Human Macrophages. 4-parameters regression of standard curve for human IL-4 (R-squared = 0.973), IL-6 (R-squared = 0.980), IL-10 (R-squared = 0.970), and TNF (R-squared = 0.917).

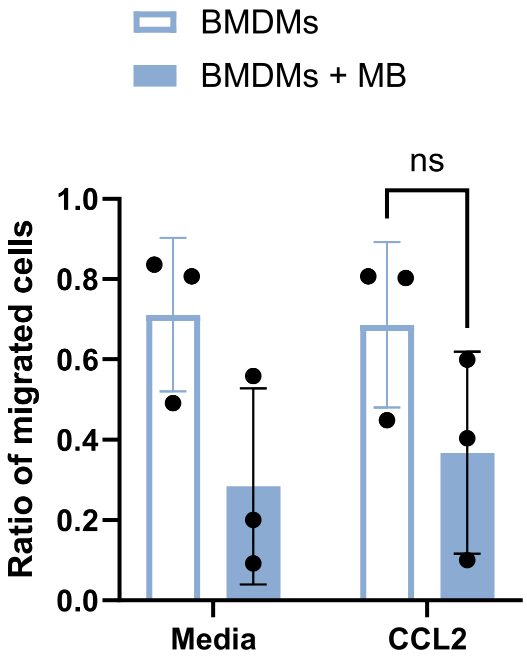

Supplementary Figure 5. Transwell migration assay (pore size = 5um) comparing migration abilities of labeled and unlabeled BMDMs (n=3 animals). Data are presented as mean values +/- SD. P values were determined by using two-way ANOVA followed by Tukey's multiple comparison. n.s. – not significant.

US imaging with AMPI (Amplitude Modulation Pulse Inversion) technique

To best implement the AMPI method we tested two methods of linear signal cancellation experimental (Fig. 1 (a) and Suppl. Fig 1 (a)-(b)). In these methods the US signals were combined in the radio frequency domain from the basis pulses to form non-linear images as follows:

$$\text{AMPI (Half Amplitude)} = P_{1.0} + P_{0.5} + P_{0.5}$$

$$\text{AMPI (Even-Odd)} = P_{1.0} + P_{even} + P_{odd}$$

Here, $P_{1.0}$ represents the negative polarity full amplitude basis pulse, and the half amplitude pulses were formed using the half amplitude basis pulse ($P_{0.5}$), the basis pulse formed using only the even elements of the transducer (P_{even}) and the basis pulse formed using only the odd elements of the transducer (P_{odd}). We observed that the sum of two half amplitude basis pulse ($P_{0.5}$) exhibited higher energy at lower frequencies compared to the full amplitude basis pulse (Suppl. Fig. 1(c)), which also resulted in incomplete vessel cancelation in the final image (Suppl. Fig. 1(d)). Conversely, the even-odd technique showed effective linear signal cancellation (Suppl. Fig. 1(b) and (c)). As a result, in our experiments we decided to employ AMPI ultrasound imaging using the even-odd technique.

Suppl. Figure 6

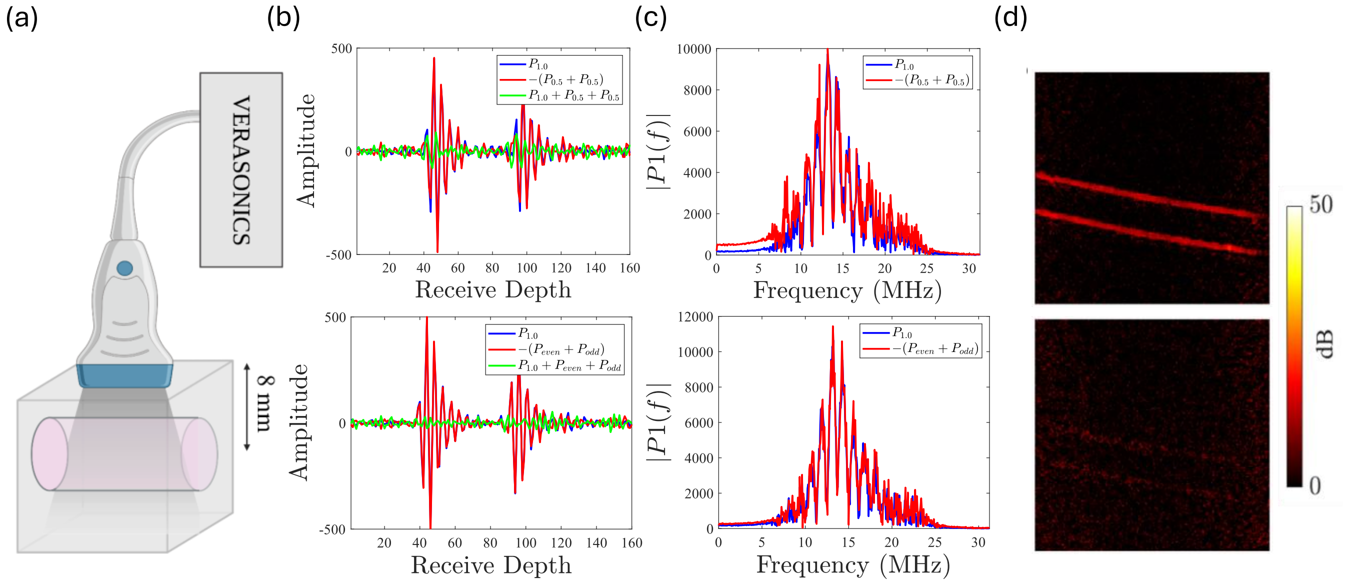

Supplementary Figure 6. Comparison of half amplitude vs even-odd technique for linear signal cancellation in AMPI. (a) Schematic of the experimental setup employed to assess linear signal cancellation between the linear interface of wall-less vessel phantom made of gelatin and filled with degassed water. “Created in BioRender. Kim, C. (2025) https://BioRender.com/1bvs0p2”. (b) Assessment of linear signal cancellation using half amplitude and even-odd technique. The comparison of the receive data from the full amplitude basis pulse, the sum of the two half amplitude basis pulses and the final processed AMPI signal using both half amplitude and even-odd technique shows residual AMPI signal in the half-amplitude technique. (b) The frequency domain comparison of the receive data from the full amplitude basis pulse, the sum of the two half amplitude basis pulses and the final processed AMPI signal using both half amplitude and even-odd technique shows higher energy at lower frequency in the half amplitude technique. (c) The image shows a cross-sectional US AMPI image of the vessel phantom with the US array arranged parallel to the flow using both half amplitude (top) and even-odd (bottom) technique.

Ultrasound signal quantification

We imaged and compared in vitro the signals of non-labeled MΦs and MB-MΦs. Briefly, control cells and MB-MΦ were mixed with low-melting-point agarose maintained at 37 °C, and poured into a pre-made well in an agarose phantom. The solution was solidified in a 4° C fridge for 5 minutes. The control cells were then imaged with US imaging array with central frequency of 15.625 MHz using B-Mode which is a linear US imaging scheme, and a combined amplitude modulation and pulse inversion (AMPI; using even-odd technique shown in Fig. 1 (a)), which is a dedicated pulse sequence for nonlinear MB imaging. For the control well and the well of interest, the SNR was quantified by measuring the average signal in an ROI and dividing it by the standard deviation of the signal in the phantom agarose (no cells, only noise).

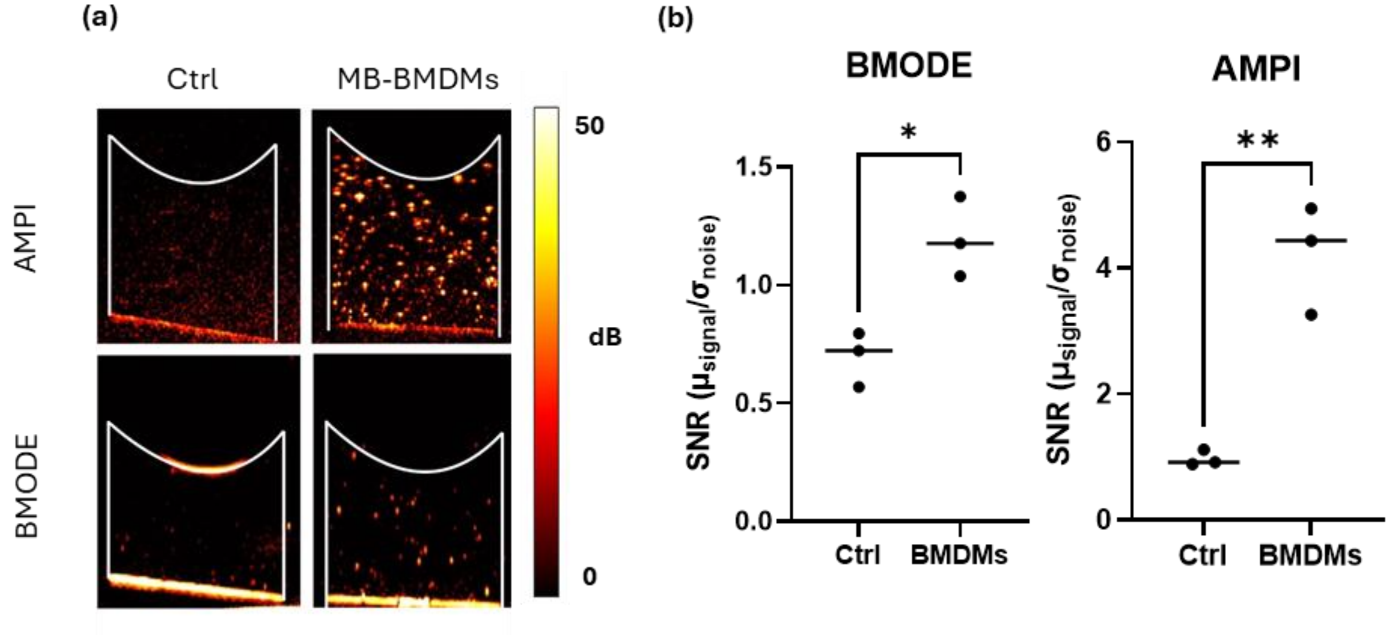

Supplementary Figure 7. (a) US imaging with BMODE and AMPI modalities of, on the left, an agarose phantom populated with unlabeled BMDMs and, on the right, a well populated with MB-BMDMS. For all phantom, 2.5k cells were mixed with low melting point agarose. **(b)** Comparisons between a phantom populated with MB-BMDMs and a phantom populated with control cells. Data are presented as mean values (horizontal line) and data points. P-values were computed with a two-tailed unpaired t-test (n=3 different ROIs, p-value = 0.003 for AMPI and p-value = 0.0134 for B-Mode). *P<0.05, **P < 0.01; n.s. - not significant.

Calibration of the US imaging array

Prior to imaging, we calibrated the US transducer (Vermon, France) using a calibrated hydrophone (Onda, Sunnyvale, CA, USA) with a reported uncertainty of ±10% in the frequency range of the excitation. The calibration for the entire operation range of the Veronicas imaging system are shown in Suppl Fig. 2.

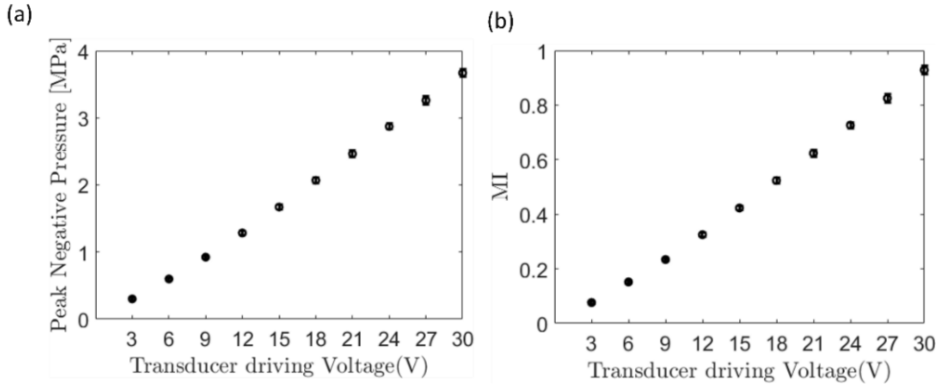

Supplementary Figure 8. **Mechanical Index derivation for L22-14V Transducer with Verasonics Vantage 256** (a) Peak negative pressure in MPa for the input voltages employed (b) Subsequent Mechanical Index or MI for the input voltages emplozed. Data are presented as mean values +/- SD.

Imaging of intratumoral injection of MΦ

The intratumoral delivery of the MB-MΦ in 4T1 murine breast cancer grown in the flank was monitored using US imaging. In these experiments, the L22-14v transducer fixed to the plane where the needle intensity was maximum. Then 200 µl of MΦ of MB-MΦ were then injected into the tumor via infusion at the rate of 100 µl/min for 2 minutes with a concentration of 1×10^7 MB-MΦ/ml for the data sown in Fig. 4(b) and 1×10^6 MB-MΦ/ml for the data shown in Fig. 4(e). Suppl. Figure 3 shows B-Mode frames of MB-MΦ entering the tumor from the needle and leaving the injection site. The full dataset is shown in Suppl. Video 3.

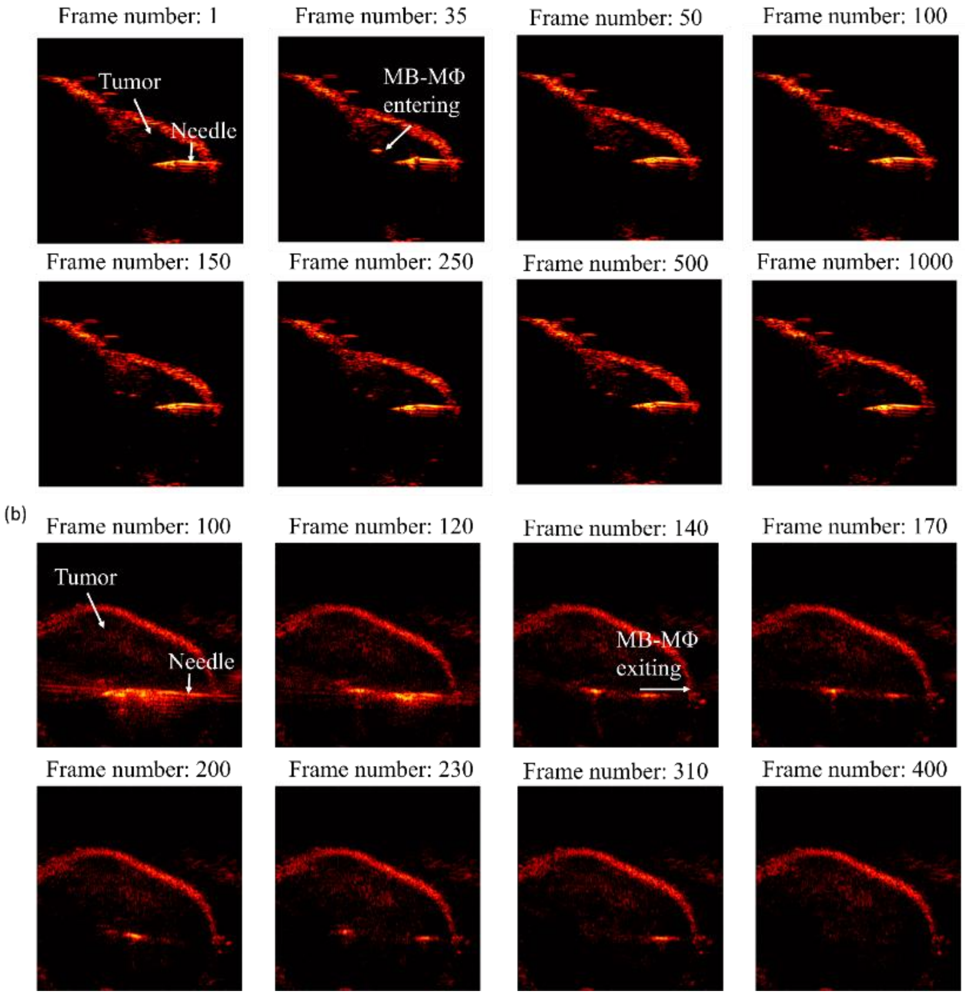

Supplementary Figure 9. Tracking MB-MΦ during and post intratumoral injection. (a) B-Mode frames showing MB- MB-MΦ entering the tumor from the needle. (b) B-Mode frames showing MB-MΦ leaving the injection site. This animal was removed from any statistics.

Vasculature mapping with MB-enhanced US *in vivo*

Supplementary Figure 4 shows the framework employed for vascular imaging following intravenous administration of MB contrast agent

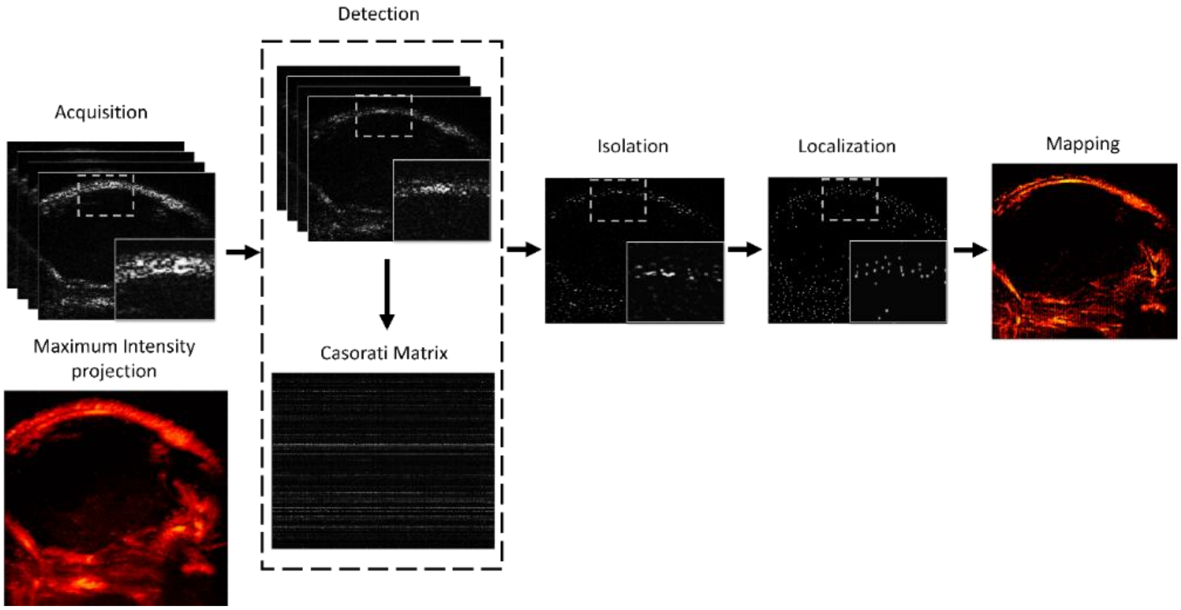

Supplementary Figure 10. Vasculature mapping with MB-enhanced US *in vivo*. (a) Framework employed for vascular imaging.

Multilevel assessment of MB-MΦ accumulation in solid tumors

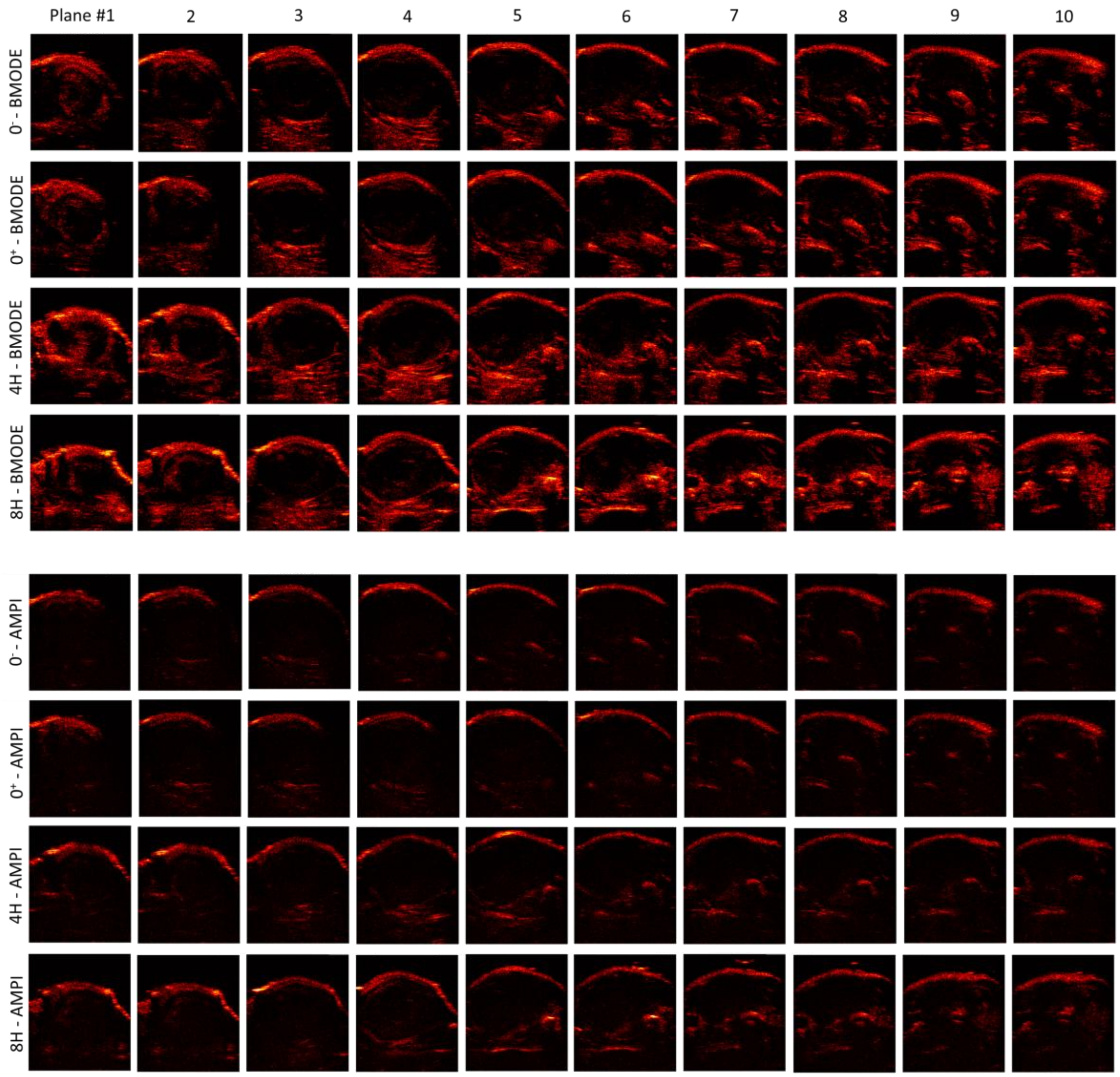

Supplementary Figure 11. Cross-sectional images of the entire tumor (Planes 1-10) following image registration at different time points following intravenous administration of MB-MΦ. Top: B-Mode frames showing MB-MΦ accumulation in the tumor as a function of time (from top to bottom). Bottom: AMPI of the same frames showing MB-MΦ accumulation in the tumor as a function of time (from top t- bottom). The images have been registered using the registration algorithm described in methods.

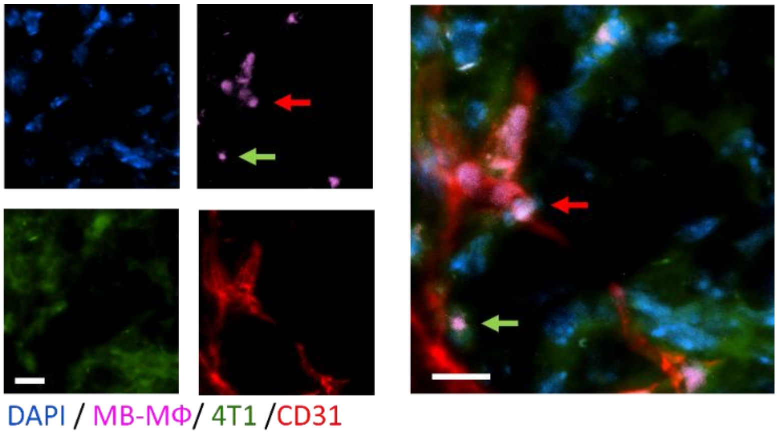

Supplementary Figure 12. Fluorescent images showing MB-MΦ (magenta) accumulation in the TME (green – cancer cells) 8 hours post intravenous administration. Red arrow indicates MΦ inside the vessel and green arrow indicates extravasated MΦ. Scale bar 100 µm.

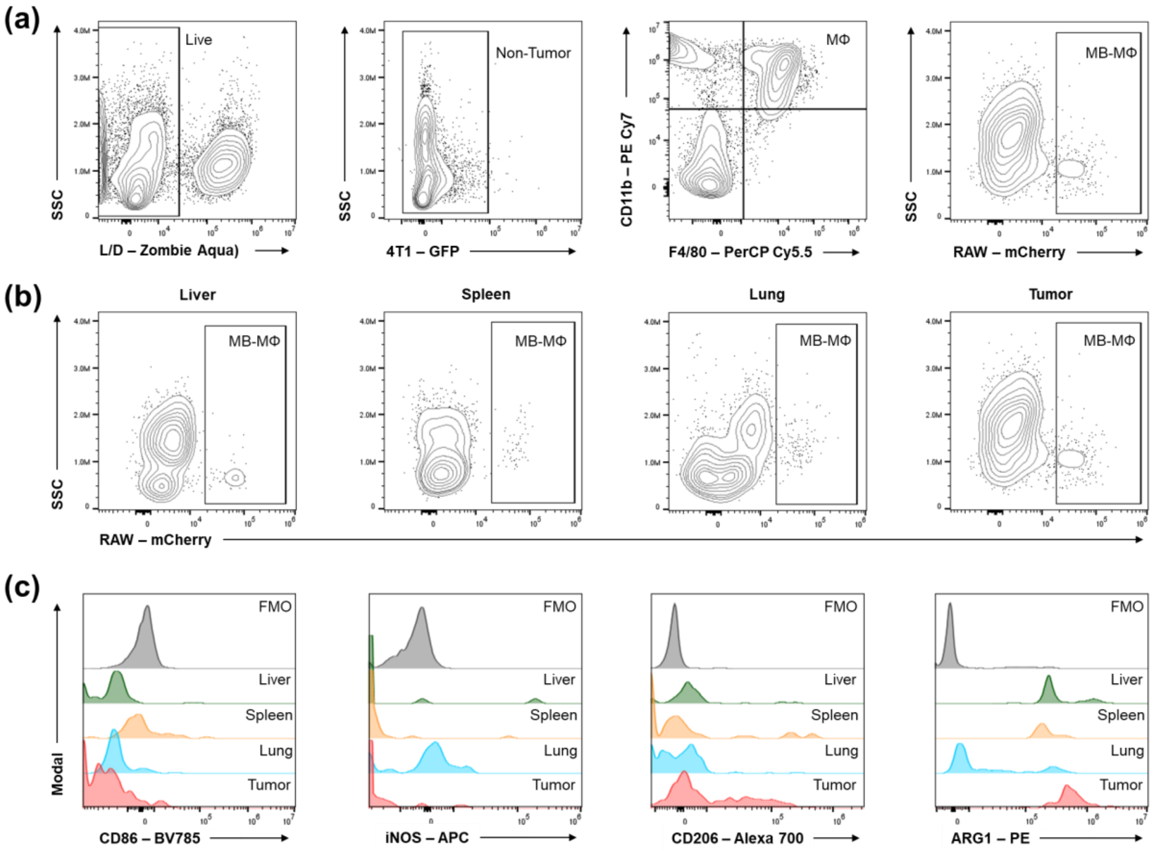

Supplementary Figure 13. Representative plots obtained from flow cytometry (in vivo) (a) Gating strategies for flow analysis of MB-labeled macrophages (MB-MΦ) *in-vivo*. **(b)** Representative MB-MΦ counts compared to host macrophages in each tissue (liver, spleen, lung, and tumor). **(c)** Representative Fluorescence Minus One (FMO) controls for macrophage phenotype markers (CD86, iNOS, CD206, and ARG1) on MB-labeled RAW 246.7 cells.

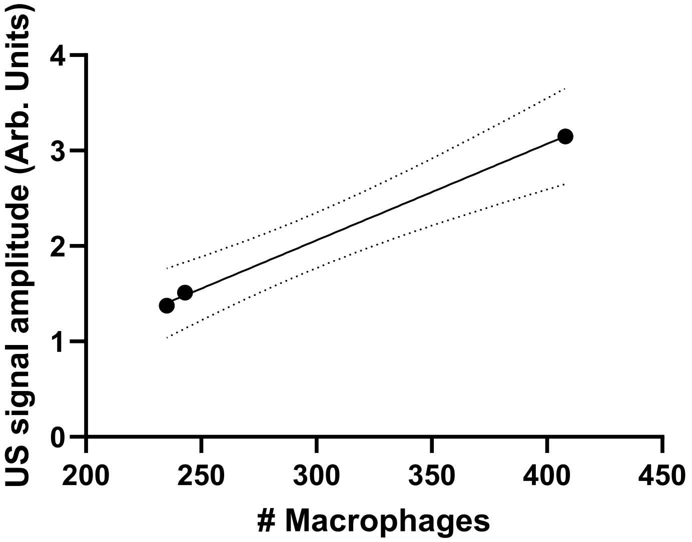

Supplementary Figure 14. Correlation analysis of exogenous macrophage infiltration and host macrophage presence within tumor tissues. Linear regression between # mCherry macrophages counted in flow and US pressure measure at 8 hours timepoint in vivo (R^2=0.9992, p = 0.018). The dashed lines represent the 95% confidence interval.

Supplementary Table 1. Properties of microbubbles used in the study

MB	Manufacturer	Gas	Shell	Marketed in
Lipid-O	In-house	C3F8	Lipid	NA
Lipid-D	In-house	C4F10	Lipid	NA
Mannose-O	In-house	C3F8	Mannose-Lipid	NA
Mannose-D	In-house	C4F10	Mannose-Lipid	NA
Definity	Lantheus Medical Imaging	C3F8	Lipid	USA, Canada, Europe, Australia, parts of Asia

Supplementary Table 2. Antibodies used in the study

	Target	Conjugate	Vendor	Catalog #	Concentration	App.
Mouse	F4/80	PerCP-Cy5.5, or BV650	Biolegend	123128, 123149	~0.5-1ug / 1x10^6 cells	FC
	CD11b	PE-Cy7, or PerCP-Cy5.5		101228, 101216	~0.3-0.5µg / 1x10^6 cells	
	CD86	BV785, or BV421		105043, 105123	~0.3-0.5µg / 1x10^6 cells	
	iNOS	APC		696808	~0.3µg / 1x10^6 cells	
	CD206	Alexa Fluor 700, or PE-Cy7		141734, 141720	~0.3µg / 1x10^6 cells	
	Arg1	PE		165804	~0.2 µg / 1x10^6 cells	
Human	CD14	Alexa Fluor 488		301811	~5µl / 1x10^6 cells	
	CD11b	PerCp-Cy5.5		393106	~5µl / 1x10^6 cells	
	CD68	APC		333810	~5µl / 1x10^6 cells	
	CD80	PE		375410	~5µl / 1x10^6 cells	
	CD163	APC-Cy7		333622	~5µl / 1x10^6 cells	
	CD1a	PE		344904	~5µl / 1x10^6 cells	
	CD83	PE-Cy7		305326	~5µl / 1x10^6 cells	
	Viability	Zombie Aqua		423102	1:500	
	CD31	Unconjugated	Abcam	ab222783	1:100	IF
	Secondary	Alexa Fluor 647	Abcam	ab150083	1:250	
	Nuclei	DAPI	Invitrogen	62248	1:1000	
