# Supplementary material for: Imaging of macrophage accumulation in solid tumors with ultrasound
Source: Nat Commun. 2025 Jul 9;16:6322. doi: 10.1038/s41467-025-61624-1 (PMC12241657; doi:10.1038/s41467-025-61624-1)
Supplement: Supplementary file 2 — Description of Additional Supplementary Files [file 41467_2025_61624_MOESM2_ESM.pdf]

Imaging of macrophage accumulation in solid tumors with ultrasound

Ashley Alva [1*], Chulyong Kim [2*], Pranav Premdas [1*], Yann Ferry [3*], Hohyun Lee [2], Nidhi Lal [3], Jing Bowen [3], Edward Botchwey [3], Brooks Lindsey [3], Costas Arvanitis [2, 3, §]

[1]Electrical and Computer Engineering, Georgia Institute of Technology, Atlanta, Georgia, United States

[2]Woodruf School of Mechanical Engineering, Georgia Institute of Technology, Atlanta, Georgia, United States

[3]Coultier Department of Biomedical Engineering, Georgia Institute of Technology and Emory University, Atlanta, Georgia, United States

*Equally contributing authors

[§] Corresponding author

Description of additional supplementary files

Supplementary Movie 1. Live cell microscopy of microbubble phagocytosis. Related to Figure 1. This time-lapse confocal microscopy video shows a RAW264.7 macrophage (expressing mCherry, magenta) actively engulfing a lipid microbubble. Following phagocytosis, the video demonstrates that the microbubble is retained within the cell, illustrating a stable labeling for subsequent imaging and tracking.

Supplementary Movie 2. High-frame rate imaging of microbubble dynamics. Related to Figure 4. This high-frame rate microscopy video (5 million frames/sec) shows the oscillation dynamics of microbubbles sonicated at a Mechanical Index of 0.21. The video compares the damped, compression-only oscillation of a microbubble that has been phagocytosed by a macrophage to the oscillation of a nearby free microbubble.

Supplementary Movie 3. Real-time ultrasound imaging of intratumoral cell infusion. Related to Figure 6. This video shows ultrasound imaging recorded during the real-time, image-guided intratumoral infusion of microbubble-labeled macrophages (MB-MΦs). The video demonstrates the ability to track the delivery of cells from the needle tip into the tumor.
