# Supplementary material for: Imaging of macrophage accumulation in solid tumors with ultrasound
Source: Nat Commun. 2025 Jul 9;16:6322. doi: 10.1038/s41467-025-61624-1 (PMC12241657; doi:10.1038/s41467-025-61624-1)
Supplement: Supplementary file 6 — Reporting Summary [file 41467_2025_61624_MOESM6_ESM.pdf]

nature portfolio

Corresponding author(s): Costas Arvanitis

Last updated by author(s): Jun 12, 2025

Reporting Summary

Nature Portfolio wishes to improve the reproducibility of the work that we publish. This form provides structure for consistency and transparency in reporting. For further information on Nature Portfolio policies, see our Editorial Policies and the Editorial Policy Checklist.

Statistics

For all statistical analyses, confirm that the following items are present in the figure legend, table legend, main text, or Methods section.

n/a	Confirmed	
☐	☒	The exact sample size (*n*) for each experimental group/condition, given as a discrete number and unit of measurement
☐	☒	A statement on whether measurements were taken from distinct samples or whether the same sample was measured repeatedly
☐	☒	The statistical test(s) used AND whether they are one- or two-sided<br>*Only common tests should be described solely by name; describe more complex techniques in the Methods section.*
☐	☒	A description of all covariates tested
☐	☒	A description of any assumptions or corrections, such as tests of normality and adjustment for multiple comparisons
☐	☒	A full description of the statistical parameters including central tendency (e.g. means) or other basic estimates (e.g. regression coefficient) AND variation (e.g. standard deviation) or associated estimates of uncertainty (e.g. confidence intervals)
☐	☒	For null hypothesis testing, the test statistic (e.g. F, t, r) with confidence intervals, effect sizes, degrees of freedom and P value noted<br>*Give P values as exact values whenever suitable.*
☒	☐	For Bayesian analysis, information on the choice of priors and Markov chain Monte Carlo settings
☒	☐	For hierarchical and complex designs, identification of the appropriate level for tests and full reporting of outcomes
☒	☐	Estimates of effect sizes (e.g. Cohen's *d*, Pearson's *r*), indicating how they were calculated

Our web collection on statistics for biologists contains articles on many of the points above.

Software and code

Policy information about availability of computer code

Data collection	NIS-Elements AR 5.21.01, Matlab R2020b, Verasonics Vantage-4.8.7-2306210900
Data analysis	Matlab R2023a, GraphPad Prism 9.5.1, ImageJ2, Microsoft Excel 2016, FlowJo 10, Python 3.12

For manuscripts utilizing custom algorithms or software that are central to the research but not yet described in published literature, software must be made available to editors and reviewers. We strongly encourage code deposition in a community repository (e.g. GitHub). See the Nature Portfolio guidelines for submitting code & software for further information.

Data

Policy information about availability of data

All manuscripts must include a data availability statement. This statement should provide the following information, where applicable:

- Accession codes, unique identifiers, or web links for publicly available datasets
- A description of any restrictions on data availability
- For clinical datasets or third party data, please ensure that the statement adheres to our policy

All data needed to evaluate the conclusions in the paper are presented in the paper and/or the Supplementary Materials.

Research involving human participants, their data, or biological material

Policy information about studies with human participants or human data. See also policy information about sex, gender (identity/presentation), and sexual orientation and race, ethnicity and racism.

Reporting on sex and gender	All human samples (i.e., blood) was de-identified (i.e., all specific identifiers removed) upon being obtained, and there fore reporting on sex and gender is not possible.
Reporting on race, ethnicity, or other socially relevant groupings	All human samples (i.e., blood) was de-identified (i.e., all specific identifiers removed) upon being obtained, and there fore reporting on race, ethnicity, or other socially relevant groupings is not possible.
Population characteristics	All human samples (i.e., blood) was de-identified (i.e., all specific identifiers removed) upon being obtained, and therefore reporting on population characteristics is not possible.
Recruitment	Recruitment was performed by word of mouth among the authors' peers, which may have led to self-selection toward members of the Georgia Tech community. This is unlikely to significantly impact the results as our data is not specific to factors such as gender, race, or other demographic variables.
Ethics oversight	All research involving human participants was approved by the Georgia Tech IRB (Protocol #H22428)

Note that full information on the approval of the study protocol must also be provided in the manuscript.

Field-specific reporting

Please select the one below that is the best fit for your research. If you are not sure, read the appropriate sections before making your selection.

[x] Life sciences   [ ] Behavioural & social sciences   [ ] Ecological, evolutionary & environmental sciences

For a reference copy of the document with all sections, see nature.com/documents/nr-reporting-summary-flat.pdf

Life sciences study design

All studies must disclose on these points even when the disclosure is negative.

Sample size	No statistical methods were used to predetermine sample size. The sample sizes (n = 3–6 per group) were selected based on established practices in comparable preclinical imaging and cell-tracking studies within the field. This size was deemed sufficient to provide meaningful data and statistical significance. The statistically significant differences observed between our experimental and control groups, as detailed in the manuscript, confirm that the chosen sample sizes were adequate to support our conclusions.
Data exclusions	For in-vivo assessment of trafficking patterns, one mouse was excluded due to registration error.
Replication	All attempts at replication were successful. The primary findings from our ultrasound imaging were highly reproducible across independent experiments and consistent in both B-mode and AMPI imaging modes. The biological findings were corroborated by multiple independent methods, all of which yielded reproducible results. For example, macrophage accumulation detected by ultrasound was consistently verified across biological replicates using fluorescence microscopy and flow cytometry. Similarly, results from cell viability, migration, and ELISA assays were reproducible across experiments, as detailed in the corresponding figure legends.
Randomization	For all experiments, animals were randomly assigned into experimental groups and tumor (4T1) bearing animals were spread equally between the groups.
Blinding	Flow-cytometry and immunofluorescence data analyses were performed blinded.

Reporting for specific materials, systems and methods

We require information from authors about some types of materials, experimental systems and methods used in many studies. Here, indicate whether each material, system or method listed is relevant to your study. If you are not sure if a list item applies to your research, read the appropriate section before selecting a response.

Materials & experimental systems

n/a	Involved in the study
	x Antibodies
	x Eukaryotic cell lines
x	Palaeontology and archaeology
	x Animals and other organisms
x	Clinical data
x	Dual use research of concern
x	Plants

Methods

n/a	Involved in the study
x	ChIP-seq
	x Flow cytometry
x	MRI-based neuroimaging

Antibodies

Antibodies used	iNOS Monoclonal Antibody (CXNFT), Alexa Fluor™ 488, eBioscience™ (53-5920-82, Invitrogen), Anti-mouse CD31 (ab28364, Abcam), Zombie Aqua (423102, Biolegend), F4/80 PerCP/Cy5.5 (123128, Biolegend), F4/80 BV650 (123149, Biolegend), CD11b PerCP/Cy5.5 (101228, Biolegend), CD11b PE/Cy7 (101216, Biolegend), CD86 BV785 (105043, Biolegend), CD86 BV 421 (105123, Biolegend), iNOS APC (696808, Biolegend), Arg1 PE (165804, Biolegend), CD206 PE/Cy7 (141720, Biolegend), CD206 Alexa700 (141734, Biolegend), CD14 Alexa 488 (301811, Biolegend), CD11b PerCP/Cy5.5 (393106, Biolegend), CD68 APC (333810, Biolegend), CD80 PE (375410, Biolegend), CD163 APC/Cy7 (333622, Biolegend), CD83 PE/Cy7 (305326, Biolegend), CD1a PE (344904, Biolegend)
Validation	All antibodies have been validated and reported in the literature (see manufacturers' website for detail)

Eukaryotic cell lines

Policy information about cell lines and Sex and Gender in Research

Cell line source(s)	Eukaryotic Cell Lines<br>1) Breast cancer cell line 4T1 - purchased from Imanis Life Sciences; catalogue #: 4T1-Fluc-Neo/GFP-Puro cells<br>2) Macrophage cell lines RAW264.7 - courtesy of Krish Roy; it can be purchased from ATCC; catalogue # TIB-71.<br>Primary Cells<br>3) Bone marrow-derived macrophages (female Balb/c, #000651, The Jackson Laboratory)<br>4) Human monocytes / macrophages / dendritic cells - primary human cd14+ monocytes (isolated from blood collected from healthy donors)
Authentication	The cell lines were purchased and used within a low passage number after receipt<br>BMDMs and Human cells were confirmed by expression of markers
Mycoplasma contamination	The cell lines used in this study were not tested for mycoplasma contamination by the authors
Commonly misidentified lines<br>(See ICLAC register)	No misidentified lines

Animals and other research organisms

Policy information about studies involving animals; ARRIVE guidelines recommended for reporting animal research, and Sex and Gender in Research

Laboratory animals	All experiments used 8- to 12-week-old female Balb/c mice (JAX).
Wild animals	This study does not involve wild animals.
Reporting on sex	Animals used in studies were female since the focus of the study is not related to sex differences, using mice of a single sex can reduce biological variability.
Field-collected samples	No field collected samples were used in the study.
Ethics oversight	All animal procedures were performed according to the guidelines of the Public Health Policy on the Humane Care of Laboratory Animals and approved by the Institutional Animal Care and Use Committee of Georgia Institute of Technology.

Note that full information on the approval of the study protocol must also be provided in the manuscript.

Plants

Seed stocks	N/A
Novel plant genotypes	N/A
Authentication	N/A

Flow Cytometry

Plots

Confirm that:

- [x] The axis labels state the marker and fluorochrome used (e.g. CD4-FITC).
- [x] The axis scales are clearly visible. Include numbers along axes only for bottom left plot of group (a 'group' is an analysis of identical markers).
- [x] All plots are contour plots with outliers or pseudocolor plots.
- [x] A numerical value for number of cells or percentage (with statistics) is provided.

Methodology

Sample preparation	At the time of sacrifice, the tumors as well as the lungs, spleen, and liver were extracted and homogenized. Cells were then filtered and leukocyte populations were selected using Percoll gradient method. The collected cells were then stained with antibodies for flow analysis.
Instrument	Cytek Aurora
Software	FlowJo was used for all analyses.
Cell population abundance	~50,000 events captured from approximately 1M single cell suspension for each sample.
Gating strategy	FSC/SSC was used to identify single cells and then gating was done using corresponding fluorophore to analyze sub-groups.

- [x] Tick this box to confirm that a figure exemplifying the gating strategy is provided in the Supplementary Information.
